# Scale‐Up of Human Amniotic Epithelial Cells Through Regulation of Epithelial‐Mesenchymal Plasticity Under Defined Conditions

**DOI:** 10.1002/advs.202408581

**Published:** 2025-01-13

**Authors:** Wangping Hao, Yi Luo, Jia Tian, Yuefeng Lu, Yangyang Cui, Ying Zhang, Xiao Jin, Hongjuan Ye, Mengqi Lu, Jinjia Song, Weiqing Zhou, Wencheng Zhang, Zhiying He

**Affiliations:** ^1^ Institute for Regenerative Medicine State Key Laboratory of Cardiology and Medical Innovation Center Shanghai East Hospital School of Life Sciences and Technology Tongji University Shanghai 200123 P. R. China; ^2^ Shanghai iCELL Biotechnology Co., Ltd Shanghai 200335 P. R. China; ^3^ Shanghai Engineering Research Center of Stem Cells Translational Medicine Shanghai 200335 P. R. China; ^4^ Shanghai Institute of Stem Cell Research and Clinical Translation Shanghai 200120 P. R. China; ^5^ State Key Laboratory of Biochemical Engineering Institute of Process Engineering Chinese Academy of Sciences Beijing 100190 P. R. China; ^6^ Key Laboratory of Biopharmaceutical Preparation and Delivery Institute of Process Engineering Chinese Academy of Sciences Beijing 100190 P. R. China; ^7^ College of Chemical Engineering University of the Chinese Academy of Sciences Beijing 101408 P. R. China; ^8^ Postgraduate Training Base of Shanghai East Hospital Jinzhou Medical University Jinzhou Liaoning 121001 P. R. China

**Keywords:** 3D microcarrier expansion, epithelial‐mesenchymal plasticity (EMP), human amniotic epithelial cells (hAECs), partial epithelial‐mesenchymal transition (pEMT), single‐cell RNA sequencing (scRNA‐seq)

## Abstract

Human amniotic epithelial cells (hAECs) have shown excellent efficacy in clinical research and have prospective applications in the treatment of many diseases. However, the properties of the hAECs and their proliferative mechanisms remain unclear. Here, single‐cell RNA sequencing (scRNA‐seq) is performed on hAECs obtained from amniotic tissues at different gestational ages and passages during in vitro culture. The results showed that the proliferation of hAECs is associated with epithelial‐mesenchymal plasticity (EMP) during amniogenesis. Freshly isolated, full‐term hAECs are identified as mature epithelial cells. Once cultured in vitro, they are observed to rapidly undergo epithelial‐mesenchymal transition (EMT) and enter a partial epithelial‐mesenchymal transition (pEMT) state to regain their EMP properties and proliferation capacities. With the continuous development of EMT, hAECs eventually enter a senescent state. The addition of SB431542 and microcarrier screening enabled the effective 3D expansion of hAECs by 50 fold while maintaining the EMP status in hAECs for further proliferation. This study not only elucidated the central proliferation mechanism of hAECs during development and expansion but also optimized the in vitro culture system so that it is sufficient to generate hAECs for 50 patients from a single donor amniotic membrane.

## Graphical abstract

Freshly isolated human amniotic epithelial cells (hAECs) from full‐term amniotic membranes include mature epithelial cells as well as cells in a senescent state. During in vitro culture, hAECs undergo epithelial‐mesenchymal transition (EMT) and enter a partial epithelial‐mesenchymal transition (pEMT) state (E/M hybrid) resembling early developmental cells, acquiring epithelial‐mesenchymal plasticity (EMP) properties and proliferative capabilities. As the EMT process progresses, cells may enter a senescent state. Inhibiting the EMT process with SB431542 after cells have acquired EMP properties is beneficial for maintaining EMP properties and proliferative capacity in hAECs, thereby enhancing the yield of hAECs.

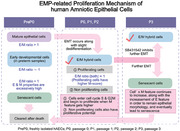

## 1 Introduction

Human amniotic epithelial cells (hAECs) have low immunogenicity and immunomodulatory abilities. They have been proven to provide a favorable microenvironment for cell survival or activate endogenous tissue regeneration and are therefore ideal candidate cells for cell therapies.^[^ ^1^, ^2^, ^3^ ^]^ However, because hAECs are presented in amniotic tissue during delivery and hAECs isolated from term amniotic tissue are at the end of amniotic development from a developmental perspective, hAECs have very limited proliferation ability. Therefore, although it is an ideal seed cell for the treatment of diseases, its application is complicated by the number of cells and the stability of the cell preparation. However, the current understanding of the properties of hAECs and the mechanisms underlying their development and proliferation during in vitro culture remains unclear. These issues present an obstacle to the widespread clinical application of amniotic epithelial cells and need to be addressed urgently.

The dynamic transformation between the epithelial and mesenchymal states of hAECs is essential to maintain the integrity of the amniotic membrane during development. This process includes epithelial‐mesenchymal transition (EMT), mesenchymal‐epithelial transition (MET), and the intermediate state (E/M), which are also involved in biological evolution, embryonic development, wound healing, fibrosis, and cancer progression. The E/M state is also called partial epithelial‐mesenchymal transition (pEMT) and is characterized by the simultaneous expression of epithelial and mesenchymal cell markers in a single cell, or by the loss of epithelial cell markers without gaining mesenchymal markers.^[^ ^4^ ^]^ The importance of unraveling the complexity and plasticity of EMT programs is evident and must be explored within a broader conceptual context.^[^ ^5^ ^]^ As a crucial component of the extraembryonic tissue, the amniotic membrane was an important milestone in the evolution of animals from aquatic to terrestrial environments. The structure of the amniotic membrane is simple and is composed of a single amniotic epithelial layer, basement membrane, compact layer, fibroblast layer, and spongy outer layer.^[^ ^6^ ^]^ It contains only hAECs in the epithelial layer and human amniotic mesenchymal cells (hMSCs) in the fibroblast layer, which represents an ideal model for revealing the role of EMT/MET in organogenesis and cell proliferation.

EMT has been shown to promote the emergence of the germ layer during animal development and is an evolutionary approach in Eumetazoa.^[^ ^7^ ^]^ In mammals, in the process of embryonic development, the sequential development of different germ layers, tissues, and organs is accompanied by the EMT and MET processes.^[^ ^8^ ^]^ EMT and MET are also involved in wound healing, fibrosis, dissociation of tumor cells, and intravasation into blood vessels.^[^ ^9^, ^10^ ^]^ Currently, studies using cell lines, developmental systems, and cancer models have revealed diverse EMT‐induced phenotypes and highlighted the remarkable complexity in the execution and regulation of EMT. However, studies on EMT remain limited to traditional developmental, cellular, and cancer biology.

The amniotic membrane has a simple structure and relatively less complicated cellular components. Amniotic cells are known to be capable of dynamically transforming between epithelial and mesenchymal states to maintain the integrity of the amniotic membrane during development.^[^ ^11^ ^]^ Therefore, the amniotic membrane is an ideal tissue for studying the role of EMT in cell fate regulation and proliferation. Inspired by the proliferation pattern of choanocytes in sponges, this study aimed to clarify the key mechanisms underlying hAECs proliferation by comparing the genetic profiles of hAECs derived from the amniotic tissue at different amniogenic stages and hAECs from different passages cultured in vitro. To clarify this proliferation mechanism, in vitro expansion of hAECs was achieved by establishing defined cell culture conditions with components that regulate the proliferation mechanism of hAECs. Alternatively, by combining hAECs with microcarriers to scale‐up, a stable quantity and quality of seed cells can be obtained for the clinical application of hAECs in the treatment of diseases.

## 2 Results

### 2.1 EMP Participated in the Development Process of hAECs In Vivo

**Figure 1:**
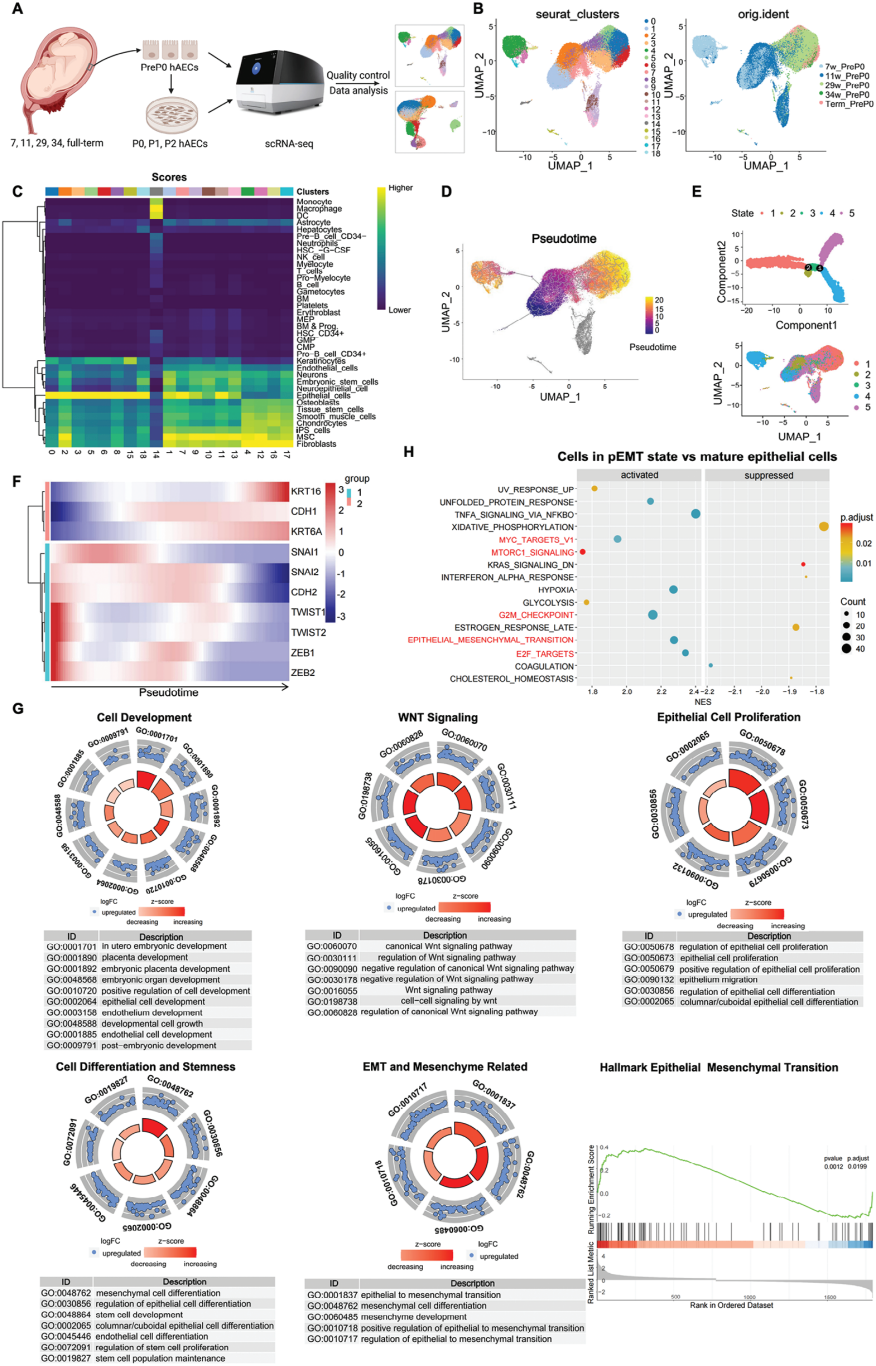
The in vivo maturation and proliferation of hAECs was related to their EMP properties. scRNA‐seq analysis of hAECs isolated from amniotic membranes (passage PreP0) of different gestational ages. A) Flowchart overview of single‐cell RNA‐seq of hAECs obtained from amniotic tissues of different gestational ages, and those from different passages during in vitro culture (Created with BioRender.com). B) Integrated UMAP plot (resolution = 0.8) of hAECs from five gestational ages (7, 11, 29, 34, and >36 weeks) with harmony. C) Annotation of subpopulations using SingleR, showing scoring results for various cell types in heatmap format. D) Pseudotime analysis of all cells with monocle 3 and mapped to UMAP plot. E) Pseudotime analysis of the filtered data with monocle 2 to obtain states representing different differentiation statuses. F) Expression trends of the biomarkers that closely related to the changes in EMP properties. G) GO enrichment analysis of differentially expressed genes between the earliest and latest states, categorization of enriched terms, and display using chord diagrams, along with supplementary evidence from GSEA of the DEGs referred above on HALLMARK_EPITHELIAL_MESENCHYMAL_TRANSITION (p‐adjust < 0.05). H) The GSEA result of differentially expressed genes between the cells in pEMT state and mature epithelial cells (when NES > 0, it is displayed as “activated”, and NES < 0 as “suppressed”).

Single‐cell RNA sequencing (scRNA‐seq) analysis was performed on hAECs from different gestational ages, including 7, 11, 29, 34 weeks, and full‐term (39 weeks), to clarify the proliferation mechanism of hAECs during development (**Figure** **1**A). After performing quality control and filtering, 19 subgroups were obtained that exhibited good cell type homogeneity, and heterogeneous subgroups emerged mainly from cell sample differences (Figure 1B). The subgroups were annotated using SingleR, and the results showed that variations between different cell subgroups were mainly due to the different intensities of epithelial or mesenchymal properties (Figure 1C). Given that the samples were obtained from amniotic tissues at different gestational ages, we speculated that the varying strengths of epithelial or mesenchymal features in the different subgroups might be due to the different developmental stages of amniogenesis.

To validate this hypothesis, we used Monocle 2 and Monocle 3 for pseudotime analysis of all cells (Figure 1D,E). The pseudotime starting point was defined according to biological developmental time, where hAECs derived from the amnions of 7th and 11th weeks (State 4, early developmental stage) were at earlier stages of development than the full‐term amnion‐derived hAECs (State 1, late developmental stage) (Figure 1E). Further analysis and validation of the expression of specific marker genes associated with EMT, which have been widely confirmed, indicated that EMT‐related markers display clear changes over time. The epithelial markers KRT16, KRT6A, and CDH1 gradually increased with pseudotime, whereas the mesenchymal markers SNAI1/2, ZEB1/2, TWIST1/2, and CDH2 showed decreased expression (Figure 1F). Based on the annotation results of SingleR (Figure 1C), and using the epithelial/mesenchymal markers and pEMT markers in the gene lists (Supplementary Material Genelist) for scoring, we found that cell clusters 0, 5, 6 (mature epithelial cells), and 18 have higher epithelial (E) attributes and lower mesenchymal (M) attributes (Figure S1A,B, Supporting Information). Moreover, most cells (clusters 1, 2, 7, 9, 10, and 13) from hAECs samples of the 7th and 11th weeks (early stage of development) had higher M attributes and lower E attributes, and the epithelial properties of the cells gradually increased with development; meanwhile, the mesenchymal properties decreased over time (Figure S1A–D, Supporting Information). In other words, hAECs in the early stages of development are in a pEMT state with epithelial‐mesenchymal plasticity (EMP) properties.

Therefore, we hypothesized that the proliferation of amniotic epithelial cells during early development is related to their EMP. Differential expression analysis was performed on the data of hAECs from the early and late stages of development, and the differentially expressed genes obtained were subjected to GO enrichment analysis. After browsing and filtering all the enriched terms, five major upregulated functional modules were obtained during early development, including cell development‐related pathways, the WNT signaling pathway, pathways related to EMT and mesenchymal development/differentiation, epithelial cell proliferation‐related pathways, cell differentiation and stem cell‐related pathways (Figure 1G). In addition, differential gene expression analysis and gene set enrichment analysis (GSEA) on cells with high M properties (clusters 1, 2, 7, 11) and high E properties (clusters 0, 3, 5, 6, 8), excluding subgroups undergoing cell cycle and those with overly strong M properties, revealed that cells with high M properties and at the early stage of development showed upregulation of EPITHELIAL_MESENCHYMAL_TRANSITION, as well as MYC_TARGETS_V1, E2F_TARGETS, G2M_CHECKPOINT, MTORC1_SIGNALING and other cell proliferation‐related signaling pathways. Thus, it was evident that during development, cells with EMP characteristics exhibited a greater proliferative ability (Figure 1H). Therefore, our findings confirmed that during embryonic development or amniogenesis, hAECs entered a pEMT state through EMT, acquiring EMP properties for their proliferation. This appears to be a key mechanism underlying the early development and proliferation of hAECs.

Next, we investigated the EMP properties of the full‐term amnion‐derived hAECs. We studied the expression patterns of the mesenchymal markers vimentin (VIM), CD90 (THY1), and CD105 (ENG), and the epithelial markers CD324 (CDH1), CK18 (KRT18), and CD9 in hAECs freshly extracted from full‐term amnions and amnions at different weeks of pregnancy (Figure S2A, Supporting Information). scRNA‐seq analysis showed that hAECs from all gestational weeks examined did not express CD105 but expressed vimentin. Those isolated from amnions at 29 and 34 weeks and at full‐term did not express CD90; hAECs from week seven showed low expression of CD324 and CD9, while full‐term amniotic epithelial cells highly expressed CD324, CD9, and another epithelial marker, CK18. The expression of epithelial and mesenchymal markers in freshly extracted full‐term hAECs was verified using qPCR (Figure S2B, Supporting Information) and flow cytometry (Figure S2C, Supporting Information), respectively. We also measured the expression levels of pluripotency‐related markers. The results indicated that hAECs freshly isolated from amnions at different weeks of gestation did not express pluripotent stem cell‐specific markers, including OCT4, NANOG, SOX2, TERT, and TERC (Figure S2D–H, Supporting Information). Other pluripotency markers, including TRA1‐81, TRA1‐60, SSEA4, and SSEA3, were detected by flow cytometry. The results showed that hAECs did not express TRA1‐81. Only 7.73 ± 6.99% of hAECs were positive for TRA1‐60. And, 62.6 ± 21.7% and 24.8 ± 11.18% hAECs were positive for SSEA4 and SSEA3, respectively (Figure S2G,H, Supporting Information). Therefore, our results confirmed that isolated full‐term hAECs were mature epithelial cells with limited pluripotency and EMP properties compared to those of early lineages.

### 2.2 Full‐Term hAECs Proliferated by Regaining EMP Feature During the In Vitro Expansion

**Figure 2:**
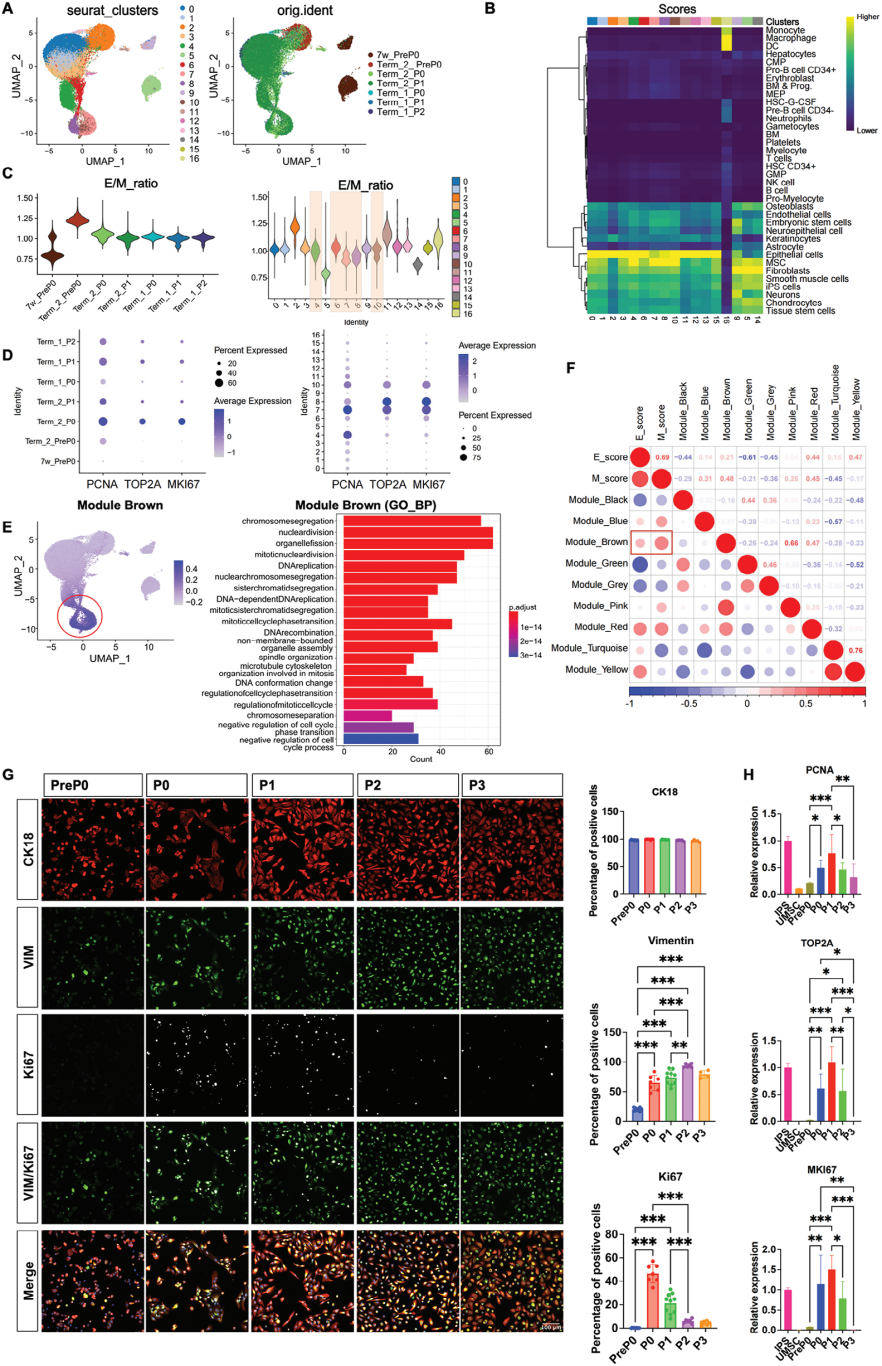
Proliferation of full‐term hAECs during in vitro culture correlated with their EMP properties. A) Integrated UMAP plot of 7 hAECs samples, including freshly isolated hAECs from 7 weeks and full‐term amniotic membranes and in vitro cultured hAECs, including P0, P1, P2 hAECs with harmony, revealing 17 subpopulations (resolution = 0.8). Term_1 and Term_2 referred to samples derived from two different amniotic tissue sources. B) Annotation of subpopulations using SingleR, showing scoring results for various cell types in heatmap format. C) The epithelial and mesenchymal scores obtained by SingleR were used to calculate the epithelial / mesenchymal ratio (E/M ratio) which was visualized by violin plots. D) Expression levels of proliferation markers (TOP2A, MKI67, PCNA) in different samples and subpopulations. E) Eight key gene modules were obtained using WGCNA, cells were scored by AddModuleScore and visualized by featureplot, and the genes in Module Brown were analyzed with GO enrichment analysis and visualized with barplot. F) The correlation coefficients of each Module with the epithelial score (E_Score) and the mesenchymal score (M_Score). G) Immunofluorescence staining was used to detect the expression of epithelial marker CK18, mesenchymal marker Vimentin, and proliferation marker Ki67 in different passages of hAECs and their statistical results. Bar = 100 µm. Data were presented as mean ± SD, and one‐way ANOVA was used for the comparison. n ≥3, *p* < 0.05 (^*^), *p* < 0.01 (^**^), and p < 0.001 (^***^). H) The expression levels of proliferation‐related genes PCNA, TOP2A, MKI67 in hAECs of different passages were detected by qPCR. Data were normalized with the positive control group set as 1 and presented as mean ± SD, and one‐way ANOVA was used for the comparison among different hAECs passages. n ≥3, *p* < 0.05 (*), *p* < 0.01 (^**^), and *p* < 0.001 (^***^).

We then analyzed the scRNA‐seq data from seven hAECs samples, including PreP0, freshly isolated full‐term hAECs, and post‐culture hAECs, namely passage 0 (P0), passage 1 (P1), and passage 2 (P2) derived from two amniotic tissue sources, labeled Term_1 and Term_2, using early developmental 7‐week amnion‐derived hAECs as references. After integration and clustering, 17 subgroups were identified (**Figure** **2**A). After cell annotation via SingleR, the macrophages were filtered for further analysis of E/M status changes (Figure 2B). Their E&M properties were assessed using epithelial and mesenchymal scores generated by SingleR, and their overall property bias was evaluated using the epithelial‐to‐mesenchymal ratio (E/M ratio). PreP0 hAECs derived from full‐term amniotic tissue exhibited an E/M ratio greater than 1, with obvious epithelial properties; after culture, their epithelial properties declined and mesenchymal properties increased, with an E/M ratio approximately equal to 1, indicating that the hAECs underwent EMT during culture, entered the pEMT state, and acquired EMP properties (Figure 2C). The expression of proliferation‐related genes (PCNA, TOP2A, and MKI67) was low in PreP0, highest in P0, and reduced in P1 and P2 hAECs compared to that in P0 hAECs (Figure 2D). This indicated that hAECs entered the pEMT state and acquired EMP properties and proliferative ability.

After observing the overall changes in the E/M ratio of the seven samples, we further analyzed the E/M properties of the subgroups. Clusters 4, 6, 7, 8, and 10 all had E/M ratios of ≈1 (Figure 2C), and exhibited stronger proliferative abilities than other cells (Figure 2D). Additionally, through weighted gene co‐expression network analysis (WGCNA), eight gene modules with high expression were identified in these cells (Figure S3A–C, Supporting Information), among which genes in Module Brown were enriched in signaling pathways related to the cell cycle and cell proliferation (Figure 2E). We performed a correlation analysis between the previously obtained E/M scores and each module. The results showed that the Brown module was positively correlated with the M score, indicating that the proliferative ability of hAECs was related to the pEMT state (Figure 2F).

Immunocytofluorescence staining analysis for the epithelial marker CK18, mesenchymal marker vimentin, and proliferation marker Ki67 was performed on hAECs at various passages. The results showed that the expression of the mesenchymal marker, vimentin, increased with cell passage. CK18 was maintained at high expression levels at all passages (Figure 2G). The cell proliferation marker Ki67 was negative for PreP0 hAECs. However, during cultivation, the Ki67 positivity increased significantly to ≈50% in P0 hAECs and subsequently declined gradually with further passages. By passages P2 and P3, Ki67 positivity levels were notably low. Interestingly, Ki67 was primarily detected in hAECs in the pEMT state (Figure 2G). Other proliferation‐related genes, PCNA, TOP2A, and MKI67, were detected using qPCR at different passages of hAECs. The results showed that the expression of all these genes exhibited the same pattern, which increased in P0 and P1 and decreased with culture passages (Figure 2H). Therefore, our results suggested that hAECs undergo EMT to enter the pEMT state and acquire EMP properties and proliferative abilities. In addition, their proliferative ability decreases with the further progression of EMT in later passages, which is the main reason for the restrained expansion of the current culture system for hAECs.

### 2.3 The E/M Ratio of hAECs Reflected the Cellular Proliferation Status, and Senescence Occurred when M Properties were Excessive

**Figure 3:**
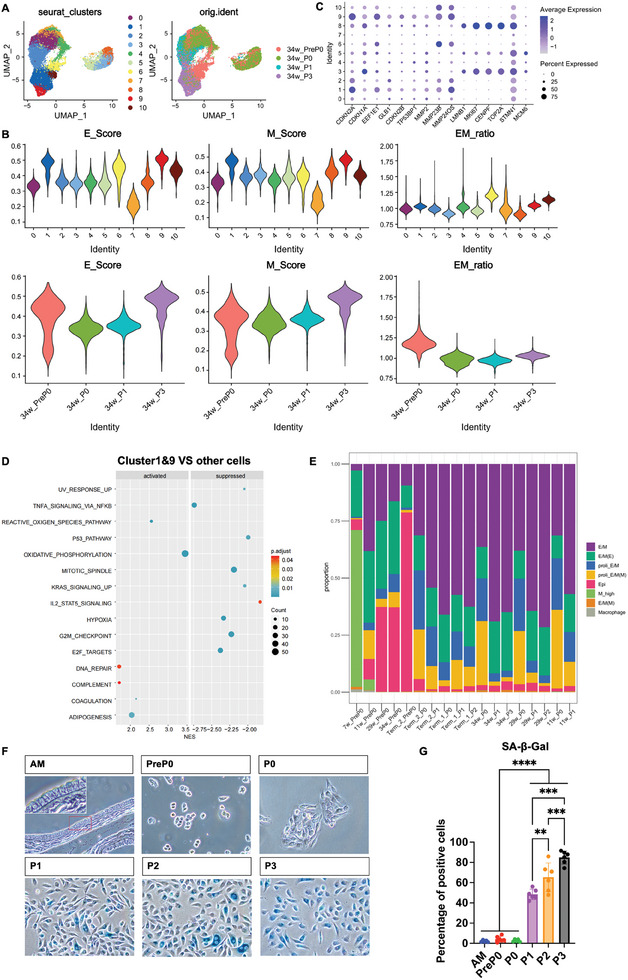
hAECs proliferated during in vitro culture in the pEMT state, and senescence occurred when mesenchymal properties were present in excess. A) Integrated UMAP plot (resolution = 0.8) of hAECs including PreP0, P0, P1, P3 isolated from amniotic membrane of 34 weeks with harmony. B) The epithelial and mesenchymal scores of different samples and cell subpopulations obtained by SingleR and the corresponding E/M ratio were visualized by violin plots. C) Dotplot depicting expression of genes related to senesence and proliferation. D) The differentially expressed genes obtained by comparing cluster 1, 9 with other cells were subjected to enrichment analysis on 50 HALLMARKs to obtain up‐regulated and down‐regulated pathways and displayed in a dot plot. E) Proportion plots showing the distribution of different subpopulations in the 18 hAECs samples. E/M, E/M (E), and E/M (M) represent cells in the pEMT state with proliferative potential, with E/M ratio close to 1, greater than 1, and less than 1, respectively. Proli_E/M and Proli_E/M(M) represent proliferating cells in the pEMT state, with E/M ratio less than 1, where the E/M ratio was lower for Proli_E/M(M). Epi represents mature cells with high epithelial properties, with E/M ratio far greater than 1. M_high represents cells with high mesenchymal properties and low epithelial properties, with E/M ratio far less than 1.F) SA‐β‐Gal senescence staining results of the amniotic membrane (AM) and the hAECs of different passages. Bar = 25 µm. G) Statistical results of SA‐β‐Gal senescence staining. Data were presented as mean ± SD, and one‐way ANOVA was used for the comparison. n ≥3, *p* < 0.05 (^*^), *p* < 0.01 (^**^), and *p* < 0.001 (^***^).

During the in vitro culture process, hAECs could not be passaged beyond three passages. After integrating and analyzing the scRNA‐seq data of hAECs from different passages derived from 34‐week amniotic tissue, we obtained 11 subgroups (**Figure** **3**A). Based on the expressions of proliferation/senescence‐related genes and cell cycle scores, we first recognized that clusters 3, 5, and 8 were proliferating cells, whereas clusters 1 and 9 were senescent cells (Figure S4A,B, Supporting Information). As shown in the unified manifold approximation and projection (UMAP) diagram of orig.ident groups in Figure 3A, the heterogeneity between P3/PreP0 and P0/P1 was large. Combined with the cell annotation results for SingleR (Figure S4C, Supporting Information), we observed that the heterogeneity of cells was mainly due to differences in epithelial and mesenchymal properties. PreP0 cells from the 34‐week gestation period showed high E/M heterogeneity (Figure 3B), overlapping with P0, P1, and P3, suggesting that PreP0 hAECs contained cells with proliferative potential (cluster 7), and mature epithelial cells (clusters 6 and 10), and senescent cells (cluster 1) (Figure 3C,D).

Further investigation revealed that cells in cluster 7 were mainly derived from P0 hAECs; both E and M properties were relatively low, and the E/M ratio was close to 1, similar to cells in early development at 7 weeks. Clusters 6 and 10 mainly came from PreP0 hAECs; they were mature epithelial cells with epithelial properties significantly stronger than mesenchymal properties, and the E/M ratio was far greater than 1 (Figure 3B; Figure S4D, Supporting Information). After culturing, P0 and P1 hAECs were divided into clusters of resting cells with proliferative potential (clusters 0, 2, and 4), cells proliferating (clusters 3, 5, and 8), and possible senescent cell clusters (clusters 1 and 9). Among them, cells proliferating after culture clustered together with cluster 7, maintaining a balanced E/M property. The proliferating cells had slightly higher M properties than resting cells, demonstrating that hAECs underwent EMT during in vitro culture and entered a pEMT state, thus acquiring EMP properties and proliferation ability similar to hAECs in early development.

The E/M ratio of hAECs from different passages also showed that with culture passaging, the E/M ratio gradually decreased, the cells underwent EMT and entered a pEMT state (Figure 3B), and cell proliferation was related to the pEMT state (Figure 3C). However, as EMT progressed, the cells underwent senescence, which was obvious at passage P3. Senescence markers such as CDKN2A (P16), EEF1E1, and GLB1 were highly expressed in P3 hAECs (clusters 1, 9), and exhibited higher E and M properties; however, their overall properties were more epithelial, and the E/M ratio was slightly greater than 1. At the same time, cell cycle‐related markers like MKI67, TOP2A, and CENPF were not expressed in clusters 1 and 9, that is, cell cycle arrested (Figure 3C). We performed differential expression analysis of cells in clusters 1 and 9 versus other cells and conducted GSEA. We found that they upregulated senescence‐related pathways such as DNA damage repair, oxidative phosphorylation, and the ROS pathway, and downregulated proliferation‐, cell cycle‐, and function‐related pathways (Figure 3D). This means that the continuous elevation of mesenchymal properties leads to cellular senescence and, ultimately, the loss of proliferative ability as the EMT process progresses.

After integrating single‐cell data from 18 hAECs cell samples derived from different weeks of gestation and culture passages and annotating the cell subgroups, we found that PreP0 hAECs derived from 29 weeks, 34 weeks, and full‐term amniotic tissues contained similar types of cells (Figure S5, Supporting Information). They all contained cell groups with proliferative potential (E/M, E/M (E), E/M (M)), some proliferating cell groups in the pEMT state (proli_E/M, proli_E/M(M)), and mature cells with high E properties (Epi). Compared to hAECs from 34 weeks gestation, full‐term amnion‐derived PreP0 hAECs had a lower proportion of cell groups with proliferative potential and proliferating cell groups in the pEMT state (Figure 3E). By staining full‐term amniotic tissue and hAECs derived from full‐term amniotic tissue with senescence‐associated β‐galactosidase (SA‐β‐Gal), the results showed that full‐term amniotic tissue, as well as hAECs of PreP0 and P0 passages, had fewer senescent cells. The proportion of senescent cells gradually increased with culture passaging, reaching ≈90% at passage P3 (Figure 3F,G). Therefore, with the increase in culture passages, the EMT process advanced, mesenchymal properties increased continuously and ultimately led to cellular senescence, which manifested as hAECs entering senescence after limited passages of proliferation.

### 2.4 Combination of CD9‐CD326 (E markers) and CD90‐VIM (M markers) Can Represent the Cellular Proliferation Status During In Vitro Expansion

**Figure 4:**
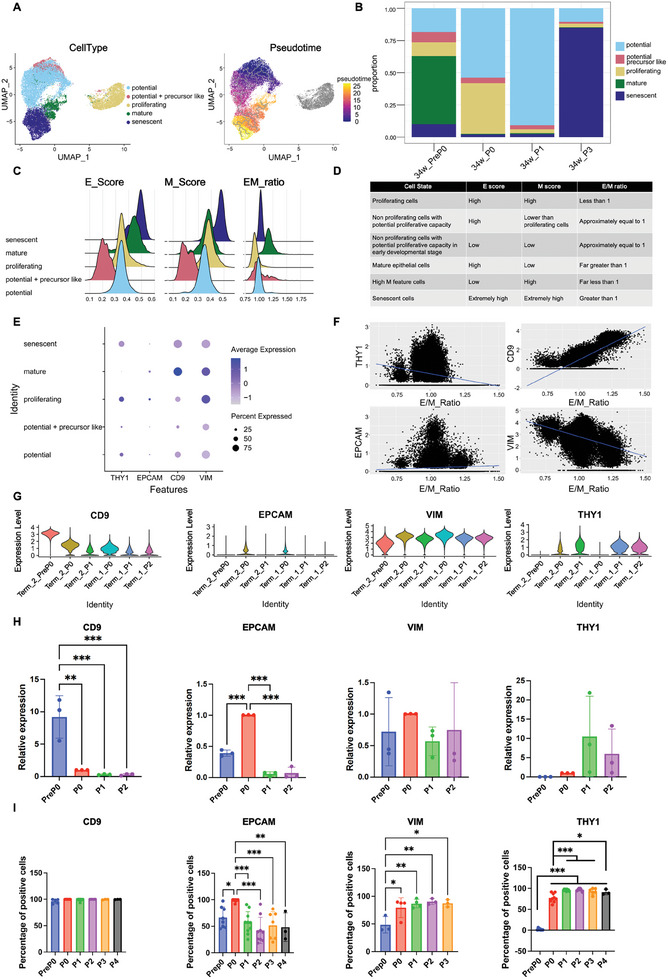
The proliferation and senescence status of hAECs during in vitro culture were associated with the cellular pEMT status. A) Annotation of different cell types based on their proliferative capacity, maturity, and senescence levels, including potential cells (cells with proliferative potential), potential+precursor like (precursor‐like cells with proliferative potential), proliferating cells (cells in a proliferative state), mature cells (mature epithelial cells), and senescent cells. Monocle 3‐based pseudotime analysis of all cells and the results were visualized on the UMAP plot. B) Proportion plot showing the distribution of different cell types in the samples. C) Ridge plots depicting epithelial, mesenchymal scores, and E/M ratio scores for different cell types. D) Summary of the relationship between E, M, E/M scores and cell status. E) Expression of four epithelial/mesenchymal‐related markers (CD9, THY1, EPCAM and VIM) in different cell types. F) A scatter plot showing the expression of CD9, THY1, EPCAM and VIM as the E/M ratio increases, with a fitted curve to visualize their expression trends. G–I) scRNA‐seq, qPCR, and flow cytometry analysis displaying the expression changes of epithelial markers CD9, CD326, and mesenchymal markers CD90, Vimentin with passage during in vitro culture. Data in Figure 4H were normalized with the passage P0 group set as 1 and presented as mean ± SD, and one‐way ANOVA was used for the comparison among different hAECs passages. Data in Figure 4I were presented as mean ± SD, and one‐way ANOVA was used for the comparison. n ≥3, *p* < 0.05 (^*^), *p* < 0.01 (^**^), and *p* < 0.001 (^***^).

Through the above assessment, we annotated the status of the cell subsets and divided them into five cell types: potential (cells with proliferative potential), potential + precursor like (cells with proliferative potential and in early development), proliferating (cells in a proliferative state), mature (mature epithelial cells), and senescent (senescent cells) hAECs (**Figure** **4**A). According to the pseudotime results from Monocle 3, there are two final states of hAECs: maturation and senescence (Figure 4A). Furthermore, the proportion plot led us to conclude that proliferating cells were predominant in passage P0, and cells with proliferative potential also existed in large numbers. Most cells in passage P1 entered the resting state, with relatively weakened proliferative capacity, and the proportion of other cells was basically consistent with that of passage P0. Mature epithelial cells and senescent cells existed in passage PreP0 at the same time, which indicated that there were two cell fates in the developmental process: one was differentiation and maturation to obtain mature hAECs with functional properties, and the other was unable to continue proliferation and enter senescence and ultimately apoptosis (Figure 4B).

Furthermore, PreP0 hAECs contained a large number of mature hAECs, a small number of senescent cells, and cells at the front end of development (Figure 4B). Combined with the E/M scoring of each cell type (Figure 4C; Figure S4D, Supporting Information), we found that, during in vitro cultivation, mature hAECs underwent EMT and entered a pEMT state. At this point, the cells were mainly divided into quiescent cells with proliferation potential and cells in a proliferative state. Both cell types exhibited balanced E/M properties. Proliferating cells exhibit enhanced mesenchymal properties. Further culture results in cells undergoing EMT with continually increasing mesenchymal properties. To maintain their epithelial cell properties, the epithelial properties also increased, and eventually, the cells entered a senescent state (Figure 4B–D). Therefore, maintaining hAECs in a pEMT state that facilitates their entry into the cell cycle by regulating the EMT process could be crucial for improving the in vitro expansion of hAECs.

To better monitor the EMP status of hAECs, we focused on CD9 and CD326 (epithelial markers) and CD90 and VIM (mesenchymal markers), which appeared in the early developmental analysis, and their gene set results of WGCNA expression patterns did not change with the analysis object. Among them, the expression peaks of CD90 and CD326 were located where the E/M ratio was equal to 1, indicating that CD90 and CD326 are likely to serve not only as mesenchymal and epithelial markers but also as indicators of the EMP state with the best proliferative capacity (Figure 4E,F). The violin plots of marker expression at different passages showed (Figure 4G) that the expression of the epithelial marker CD9 gradually decreased with culture passages, and the expression of CD326 showed a trend that first increased until passage P0 and then decreased with further culture passages, which was consistent with the trend of proliferation‐related genes (Figure 2H). Furthermore, the mesenchymal marker CD90 gradually increased, and vimentin expression slightly increased with culture passages.

The scRNA‐seq analysis results were further validated. Changes in the expression of CD9, CD326, CD90, and VIM were examined by qPCR and flow cytometry in at least three batches of full‐term hAECs during culture passaging (Figure 4H,I). The qPCR results for CD9 were consistent with those of the scRNA‐seq analysis. However, the flow cytometry results for CD9 showed a consistently high positivity rate of ≈100% upon culture passage and did not show a decreasing trend, suggesting that the decline in CD9 expression did not exceed the threshold of the flow cytometry assay within the passages we tested. The qPCR and flow cytometry results for CD326 and CD90 were consistent with those of scRNA‐seq analysis. The results of the flow cytometry assay showed that the percentage of vimentin‐positive cells gradually increased with culture passages, which is consistent with the results of scRNA‐seq analysis and immunocytofluorescence staining (Figure 2G).

### 2.5 SB431542 Maintained the EMP Properties of hAECs and Promoted Stable Cellular Proliferation

**Figure 5:**
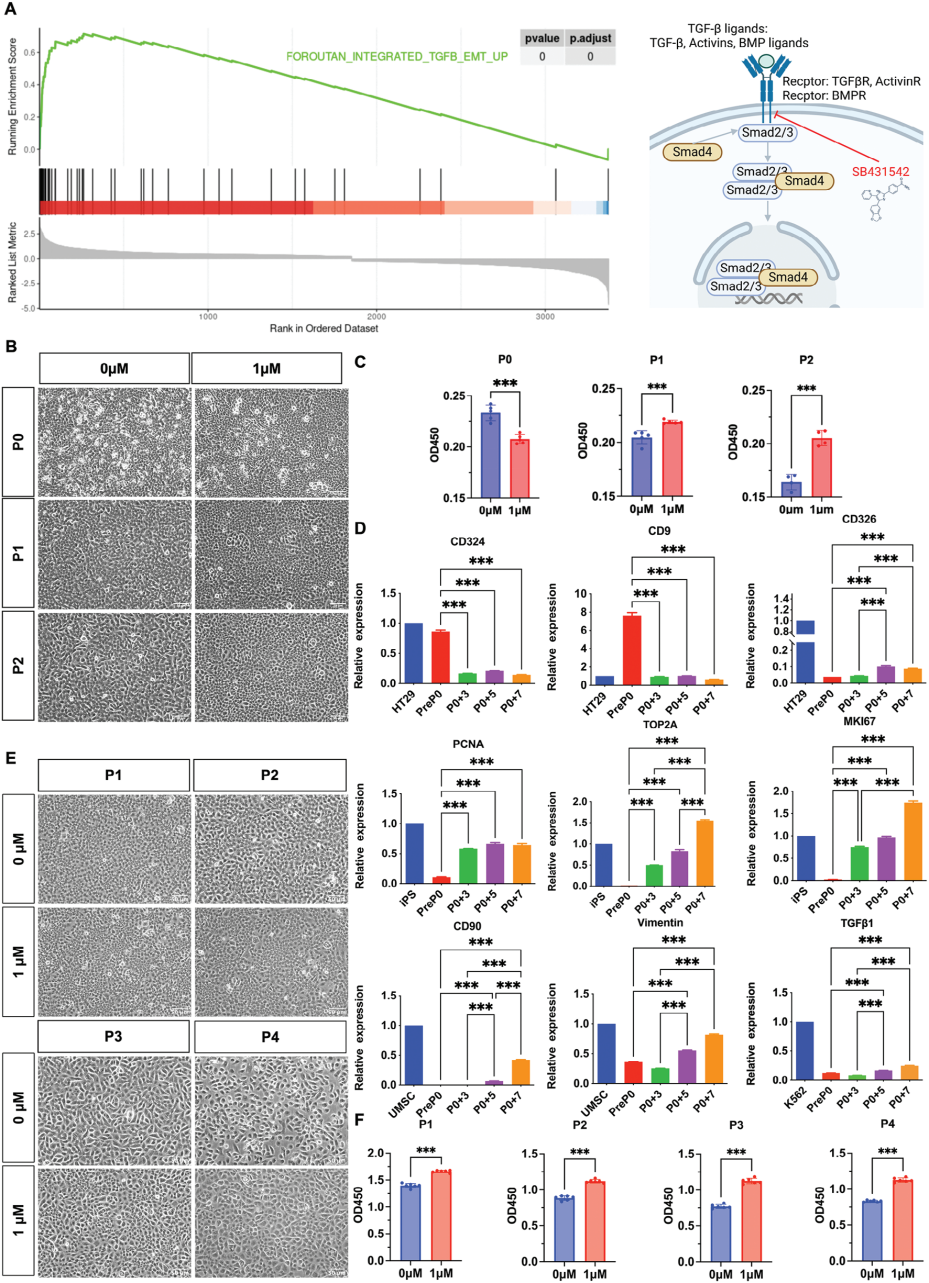
Using the TGF‐β signaling inhibitor SB431542 maintained the EMP properties of hAECs and promoted cell proliferation. A) GSEA of differentially expressed genes between proliferating cells and others on the TGFB_EMT_UP pathway (p‐adjust < 0.01), and the effect of SB431542 on the TGF‐β signaling pathway was shown (Created with BioRender.com). B) Effects of adding SB431542 from passage P0 on the morphology of hAECs in P0, P1, and P2 passages. Bar = 50 µm. C) CCK8 assay was used to evaluate the impact of adding SB431542 from passage P0 on the proliferation of hAECs in P0, P1, and P2 passages. Data were presented as mean ± SD, and unpaired two‐tailed student's t‐test was used for the comparison. n ≥3, *p* < 0.05 (^*^), *p* < 0.01 (^**^), and *p* < 0.001 (^***^). D) qPCR analysis of the expression changes of epithelial markers CD324, CD9, CD326, mesenchymal markers CD90, vimentin and TGF‐β1 and proliferation‐related genes PCNA, TOP2A, MKI67 at different culture days of passage P0. Data were normalized with the positive control group set as 1 and presented as mean ± SD, and one‐way ANOVA was used for the comparison among hAECs cultured on different days. n ≥3, *p* < 0.05 (^*^), *p* < 0.01 (^**^), and *p* < 0.001 (^***^). E) Effects of adding SB431542 from passage P1 on the morphology of hAECs in P1, P2, P3, and P4 passages. Bar = 50 µm. F) CCK8 assay was used to evaluate the impact of adding SB431542 from passage P1 on the proliferation of hAECs in P1, P2, P3, and P4 passages. Data were presented as mean ± SD, and unpaired two‐tailed student's t‐test was used for the comparison. n ≥3, *p* < 0.05 (^*^), *p* < 0.01 (^**^), and *p* < 0.001 (^***^).

GSEA enrichment analysis of differentially expressed genes in proliferating cells revealed that they were upregulated in the transforming growth factor beta (TGF‐β)‐induced EMT pathway (**Figure** **5**A). By adding SB431542, an inhibitor of the TGF‐β signaling pathway, at concentrations of 0, 2.5, 5, and 10 µM into the hAECs culture system from passage P0, we successfully maintained the cobblestone‐shaped morphology and CD326 positivity rate of hAECs (Figure S6A,B, Supporting Information). The addition of SB431542 did not affect the adherence of hAECs but had different effects on the proliferation of P0 and P1 hAECs. There was a primary inhibitory effect of SB431542 on the proliferation of P0 hAECs, while a promotion effect was observed when adding either 2.5 or 5 µM SB431542 into the P1 hAECs (Figure S6C, Supporting Information). This effect was consistent when the concentration of SB431542 was reduced to 1 µM (Figure 5B,C; Figure S7; Tables S1 and S2, Supporting Information).

Based on the above observations, we speculated that hAECs undergo EMT and enter a pEMT state before entering the cell cycle. It was verified that at passage P0, hAECs underwent EMT and entered the pEMT state, which is suitable for proliferation during in vitro culture. Specifically, we found that the expression of epithelial markers CD324, CD9, and CD326 was significantly decreased, the expression of mesenchymal markers CD90, vimentin and TGF‐β1 was significantly increased, and the expression of proliferation‐related genes PCNA, TOP2A, and MKI67 gradually increased with culture, suggesting that hAECs acquired EMP properties and gained proliferative capacity after entering the pEMT state (Figure 5D). Therefore, further experimental verification confirmed that SB431542 needed to be added starting from the P1 generation, and the concentration of 1 µM was the most favorable for proliferation culture (Figure 5E,F; Figure S8, Tables S3 and S4, Supporting Information).

To maximize the effect of pEMT on cell proliferation, we further optimized the culture conditions for hAECs. We previously developed a serum‐free medium with defined conditions that were sufficient to prepare cells for scRNA‐seq. The condition‐defined media were named MQ1 and MQ2, and MQ1 was composed of F12/DMEM, Serum Replacement, L‐glutamine, nonessential amino acid, sodium pyruvate, and EGF. MQ2 was composed of MQ1 supplemented with hydrocortisone. After proving that the benefits of adding SB431542 could further improve cell yield, we defined MQ2+SB431542 as MQ3 and focused on optimizing the cell inoculation density to maximize the total cell number obtained from a single amniotic tissue.

Different cell densities of hAECs, including 8 × 10^4^/cm^2^, 4 × 10^4^/cm^2^ and 2 × 10^4^/cm^2^ were tested. The cell morphology of passage P1 hAECs proliferating from the original cells with different inoculation densities was well‐maintained (Figure S9A, Supporting Information). The overall cell yield increased by ≈2–3 folds for 4 × 10^4^/cm^2^, and 3–5 folds for 2 × 10^4^/cm^2^ compared with that generated from 8 × 10^4^/cm^2^, respectively. The positivity rates of CD326 and vimentin, although slightly reduced in the low‐density groups, were not significantly different from those under high‐density conditions (8 × 10^4^/cm^2^) (Figure S9B,C, Supporting Information). When adding 1 µM SB431542 into the culture condition of the low density (2 × 10^4^/cm^2^), cell morphology and the expression of markers were well kept and the total cell yield of hAECs increased by ≈20% in passage 1 compared with the culture strategy based on a cell density of 2 × 10^4^/cm^2^ (Figure S9D–G and Video S1, Supporting Information).

Further analysis of hAECs using RNA‐seq analysis showed that the properties of hAECs cultured at the optimized scheme with low density (2 × 10^4^/cm^2^) in combination with SB431542 (1 µM) (P1‐2‐SB1) were highly similar to those of hAECs cultured at high density (8 × 10^4^/cm^2^) (P1‐8) (Figure S9H, Supporting Information). The prostaglandin E2 (PGE2) secretion capacity and proliferation inhibition ability of peripheral blood mononuclear cells (PBMCs) were also retained in hAECs prepared using the optimized procedure (Figure S9l,J, Supporting Information). No changes in karyotypes or abnormal karyotypes were identified in hAECs expanded in the culture system containing SB431542 (Figure S9K, Supporting Information). In addition, no significant differences in the stress response, expression of senescence‐associated genes, and proliferation‐related genes compared to P1‐8 hAECs, while the TGF‐β pathway was downregulated in P1‐2‐SB1 hAECs. (Figure S10, Supporting Information). Overall, this condition‐defined serum‐free MQ3 medium, together with low cell density, enabled 29 to 48 fold proliferation of hAECs at P1, which was 3.6 to 6 fold higher than that of the conventional 2D culture system.

### 2.6 Scale‐Up of hAECs with the 3D Microcarriers

**Figure 6:**
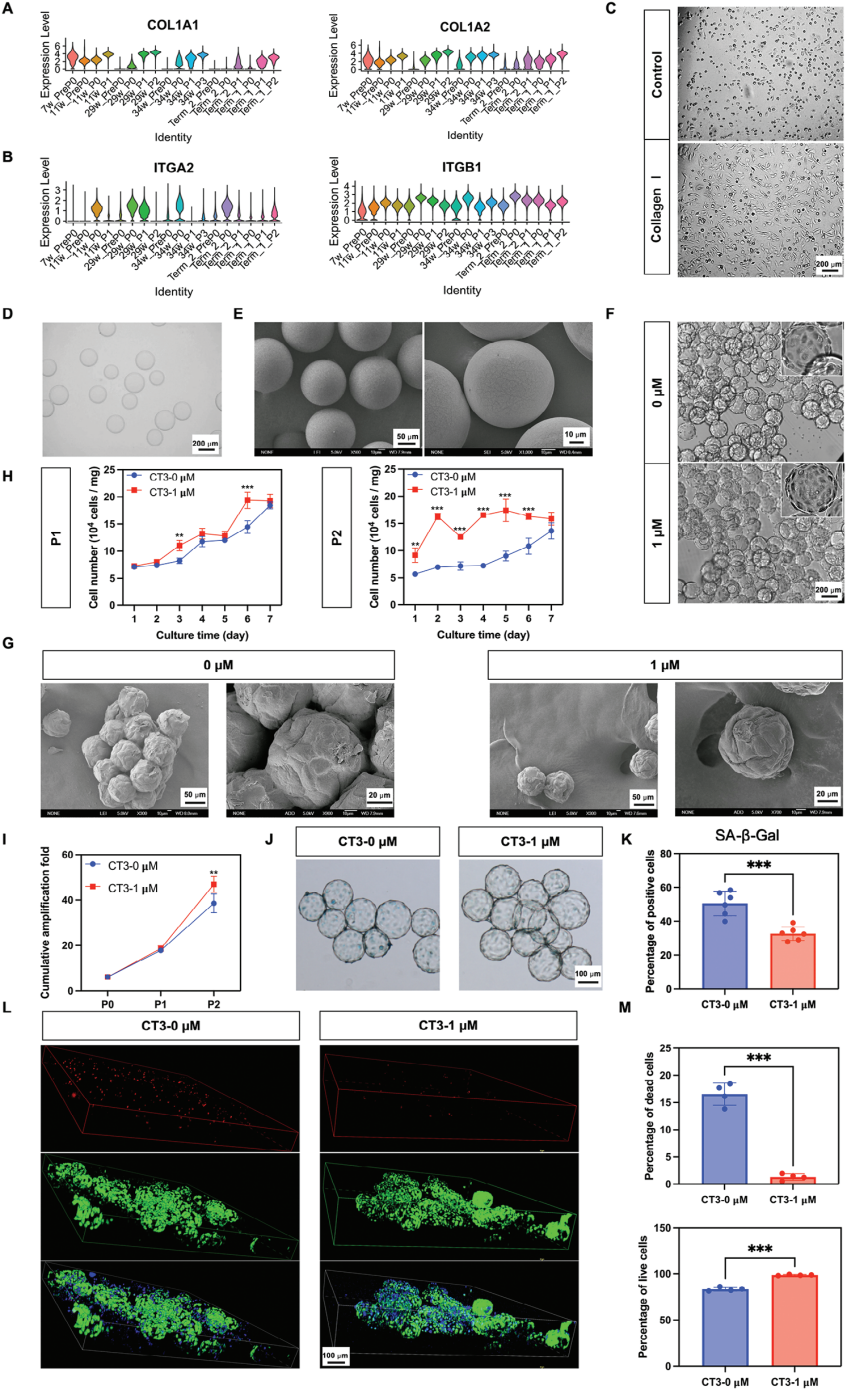
Scale‐up of hAECs with type I collagen coated microcarriers. A) The expression of type I collagen subunits in hAECs were analysed by scRNA‐seq. B) The expression of type I collagen receptor, integrin α2β1, in hAECs were analysed by scRNA‐seq. C) The influence of type I collagen on the adhesion of hAECs. Bar = 200 µm. D, E) Optical microscopy and scanning electron microscopy (SEM) images of CT3 microcarriers. Bar = 200 µm in optical microscopy images, Bar = 50 and 10 µm respectively in SEM images. F) Optical microscopy showed the morphology of hAECs on CT3 microcarriers with or without SB431542. Bar = 200 µm. G) SEM showed the morphology of hAECs on CT3 microcarriers with or without SB431542. Bar = 50 and 20 µm respectively. H) Growth curve showed the influence of SB431542 on the proliferation of hAECs on CT3 microcarriers in passages P1 and P2. Data were presented as mean ± SD, and two‐way ANOVA was used for the comparison. n  = 3, *p* < 0.05 (^*^), *p* < 0.01 (^**^), and *p* < 0.001 (^***^). I) The cumulative amplification fold of hAECs on CT3 microcarriers with or without SB431542. Data were presented as mean ± SD, and two‐way ANOVA was used for the comparison. n  = 3, *p* < 0.05 (^*^), *p* < 0.01 (^**^), and *p* < 0.001 (^***^). J) SA‐β‐Gal senescence staining results of hAECs in passage P1 cultured in on 3D CT3 microcarriers with or without SB431542. Bar = 100 µm. K) The statistical results of SA‐β‐Gal senescence staining. Data were presented as mean ± SD, and unpaired two‐tailed student's t‐test was used for the comparison. n ≥3, *p* < 0.05 (^*^), *p* < 0.01 (^**^), and *p* < 0.001 (^***^). L. Cell viability was examined by live/dead staining, where live cells were in green and dead cells were in red. Bar = 100 µm. M) The statistical results of live/dead staining. Data were presented as mean ± SD, and unpaired two‐tailed student's t‐test was used for the comparison. n ≥3, *p* < 0.05 (^*^), *p* < 0.01 (^**^), and *p* < 0.001 (^***^).

Finally, we optimized the expansion system of hAECs from a 2D attachment culture to a 3D‐microcarrier‐based system, which enabled further scale‐up of the preparation of hAECs based on a culture system consisting of a low cell density (2 × 10^4^cm^2^) combined with MQ3. Based on scRNA‐seq analysis, we found that hAECs in early development and in the cultured full‐term hAECs express high levels of Type I collagen subunits COL1A1 (7 weeks, 11 weeks, P1, P2) and COL1A2 (7 weeks, 11 weeks, P0, P1, P2) (**Figure** **6**A). The α2 subunit ITGA2 of Type I collagen receptor integrin α2β1^[^ ^12^ ^]^ was highly expressed in hAECs of passage P0, while the β1 subunit ITGB1 showed high expression in all samples (Figure 6B). Because hAECs presented a significant advantage in proliferation at passages P0 and P1 (Figure 2G,H), we speculated that type I collagen may favor the proliferation of hAECs. By culturing hAECs on Type I collagen‐coated cell culture plates, we observed a significant increase in the number of attached hAECs on the coated surface compared to that on non‐treated surfaces (Figure 6C).

To scale‐up hAECs with microcarriers, we tested several commercial microcarriers, including Cytodex 1 and Cytodex 3 (Cytiva). Cytodex 1 (CT1) microcarriers are positively charged and are formed by a cross‐linked dextran matrix substituted with positively charged N,N‐diethylaminoethyl (DEAE) groups to a degree that is optimal for cell growth. Cytodex 3 (CT3) is a microcarrier formed by a cross‐linked dextran matrix coated with a thin layer of acid‐denatured type I collagen. Our results showed that hAECs attached well to the surfaces of both CT1 and CT3 microcarriers (Figure S11A,B, Supporting Information). However, hAECs cultured on CT3 microcarriers exhibited better proliferation (Figure S11C, Supporting Information) while maintaining their epithelial properties (Figure S11D,E, Supporting Information). Optical microscopy (Figure 6D) and scanning electron microscopy (Figure 6E) revealed that CT3 presented a smooth surface where hAECs retained their typical cobblestone‐shaped morphology (Figure 6F). Cell morphology was significantly enhanced upon changing to MQ3 or adding SB431542 to MQ2 (Figures 6F,G). Specifically, after culturing the microcarrier‐loaded hAECs with MQ3, we found that SB431542 significantly promoted the proliferation of hAECs at passages P1 and P2 (Figure 6H) and dramatically increased the fold expansion of hAECs at passage P2 (Figure 6I). Interestingly, we found that SB431542 in MQ3 alleviated cellular senescence (Figure 6J,K) and maintained better cell viability (Figure 6L,M) on CT3 microcarriers. By combining MQ3 with CT3 within three passages, we were able to achieve 50 fold expansion of primary hAECs within a month (Figure 6I).

**Figure 7:**
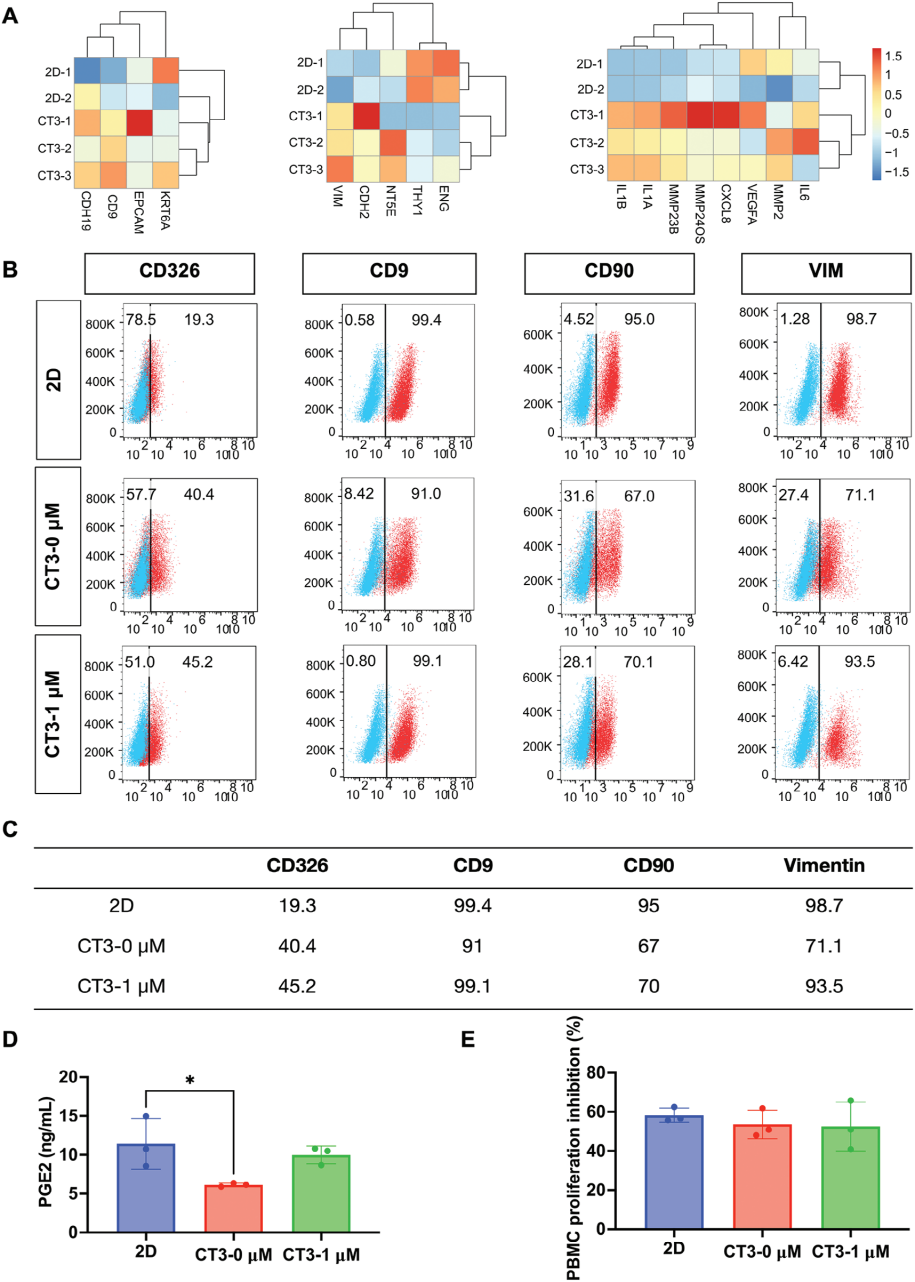
The impact of 3D cultivation on the properties of hAECs. A) RNA‐seq analysis were used to assess the impact of CT3 microcarriers on the properties of hAECs. B,C) Expression of epithelial and mesenchymal markers in hAECs cultured on CT3 microcarriers were analysed by flow cytometry. D) The PGE2 secretion ability of hAECs after culturing them on 2D culture plates and 3D CT3 microcarriers, with or without SB431542. Data were presented as mean ± SD, and one‐way ANOVA was used for the comparison. n  = 3, *p* < 0.05 (^*^), *p* < 0.01 (^**^), and *p* < 0.001 (^***^). E) The ability of hAECs to inhibit PBMC proliferation was assessed after culturing them on 2D culture plates and 3D CT3 microcarriers, with or without SB431542. Data were presented as mean ± SD, and one‐way ANOVA was used for the comparison. n  = 3, p < 0.05 (^*^), *p* < 0.01 (^**^), and *p* < 0.001 (^***^).

Scale‐expanded hAECs were further characterized by their stable cellular properties using RNA sequencing (**Figure** **7**A) and flow cytometry (Figure 7B,C). The results indicated that hAECs on CT3 microcarriers exhibited enhanced epithelial properties and increased secretion of MMP2, VEGFA, and other cytokines. Moreover, the addition of SB431542 improved the preservation of the epithelial characteristics of hAECs, while inhibiting mesenchymal traits. Additionally, we investigated the functional properties of hAECs through PGE2 secretion and inhibition of PBMC proliferation and found that although the secretion of PGE2 on the surface of microcarriers was lower, the addition of SB431542 increased its secretion to a level comparable to that of hAECs cultured in culture plates. Furthermore, hAECs from different groups exhibited similar inhibitory effects on PBMC proliferation (Figure 7D,E). Therefore, our results proved that by rationally regulating the EMP properties of hAECs, stable and scaled‐up hAECs culture and providing sufficient hAECs for their clinical applications could be achieved.

## 3 Discussion

Human amniotic epithelial cells (hAECs) have shown advanced therapeutic effects in various diseases, owing to their low immunogenicity, high tissue compatibility, non‐tumorigenicity, strong paracrine function, and immunomodulatory effects. However, the limited proliferation ability and unclear proliferation mechanism set boundaries for the further expansion of clinical applications.^[^ ^13^ ^]^ Numerous studies have found that hAECs express pluripotency markers such as OCT4, NANOG, SOX2, TRA1‐60, and TRA1‐81, indicating their multidirectional differentiation potential.^[^ ^3^, ^14^, ^15^ ^]^ However, it is unreasonable for hAECs, which are terminally differentiated cells that do not express telomerase,^[^ ^14^ ^]^ to possess pluripotency. To clarify the understanding of hAECs, in this study, we used scRNA‐seq to analyze hAECs from different gestational ages and found that early‐stage proliferation of hAECs relied on their epithelial‐mesenchymal plasticity (EMP). In contrast, hAECs from termed tissues are mature epithelial cells with no EMP features and limited proliferation capacity. Once cultured in the expansion system, hAECs can initiate the EMT process to regain their EMP features and achieve expansion. By further establishing an E/M ratio tool, the proliferation status of hAECs at different passages could be precisely retrieved and evaluated, which led us to develop optimized expansion schemes for hAECs by reducing the seeding density of hAECs and using 3D microcarriers supplemented with EMP‐retaining small molecules (i.e., SB431542).

### 3.1 Properties of hAECs

Similar to a previous report,^[^ ^14^ ^]^ we found that hAECs isolated from full‐term amniotic tissues do not express telomerase and have no telomerase activity (Figure S2, Supporting Information), but have some proliferation capacity. In this study, scRNA‐seq analysis of hAECs isolated from amniotic tissues at different gestational ages revealed that full‐term amnion‐derived hAECs were mature epithelial cells without EMP properties (Figure S2, Supporting Information). To validate the results of our scRNA‐seq analysis and define hAECs‐specific markers, we performed qPCR, flow cytometry, immunofluorescence detection, and analyzed isolated full‐term amniotic tissue hAECs in at least three duplicates, focusing on their EMP properties and pluripotency. The results showed that telomerases TERT and TERC were not expressed in hAECs; the classical pluripotent markers OCT4, NANOG, SOX2, and TRA1‐81, and the mesenchymal cell markers CD90 and CD105 were not expressed, but the classical epithelial cell markers CD324, CK18, and CD9 were expressed. The experimental results confirmed our conclusion.

During proliferation in vitro culture, hAECs undergo EMT, the expression of epithelial cell markers decreases, the expression of mesenchymal cell markers increases, and cell morphology changes from a typical cobblestone‐shaped appearance to a mesenchymal‐like appearance.^[^ ^16^ ^]^ Under existing culture conditions, the proliferation of hAECs is limited and it is difficult to exceed the P7 passage.^[^ ^17^ ^]^ Compared with the hAECs of PreP0, the telomere length of passage P5 hAECs is slightly shorter, but still belongs to the long telomere range,^[^ ^18^ ^]^ indicating that under suitable in vitro culture conditions, the proliferation ability of hAECs has room for improvement.

### 3.2 Proliferation Mechanism of hAECs

Life has evolved from unicellular to multicellular stages, after which organisms have gradually evolved into more advanced and complex individuals. Sponges are the most primitive multicellular animals, the last common ancestor of all living animals today, and do not form tissues or organs.^[^ ^19^ ^]^ In 2019, the research group of Bernard M. Degnan and Sandie M. Degnan compared the transcriptomes, fates, and behaviors of the three primary sponge cell types ‐choanocytes, pluripotent mesenchymal archaeocytes, and epithelial pinacocytes, with the choanoflagellate *Salpingoeca rosetta*, the filasterean *Capsaspora owczarzaki* and the ichthyospores *Creolimax fragrantissima* and found that, in sponges, choanocytes with epithelial cell function can transform into pluripotent mesenchymal archaeocytes through which proliferation and differentiation are achieved.^[^ ^20^ ^]^ This process is similar to EMT and MET during embryonic development in animals, with a higher degree of evolution.

EMT is a process by which epithelial cells transform into a mesenchymal state under certain physiological and pathological conditions.^[^ ^8^ ^]^ EMT is a continuous process in which cells exhibit epithelial (E), intermediate (EM), and mesenchymal (M) phenotypes.^[^ ^21^ ^]^ The EM state is also called partial epithelial‐mesenchymal transition (pEMT) and is characterized by the simultaneous expression of epithelial and mesenchymal cell markers in a single cell, or by the loss of epithelial cell markers without gaining mesenchymal markers.^[^ ^4^ ^]^ Cells in the pEMT state can switch through different states along the EMT spectrum, namely epithelial–mesenchymal plasticity (EMP),^[^ ^22^ ^]^ enabling cells to proliferate, migrate, or mature, and adapt more easily to their environment. Previous studies have confirmed that EMT occurs in hAECs during proliferation under in vitro culture conditions;^[^ ^11^, ^16^ ^]^ however, the mechanism of hAECs proliferation has not yet been explored.

EMT promotes the migration and proliferation of tumor cells.^[^ ^23^ ^]^ In our scRNA‐seq analysis, we found that hAECs were in a state of pEMT early in their development and that these cells were enriched with proliferation and stemness‐related pathways, indicating that the proliferation of hAECs during development was related to the properties of EMP. In addition, it has been reported that the stem cell characteristics of trophoblasts are related to the pEMT state,^[^ ^24^, ^25^ ^]^ and that inhibition of the EMT process is beneficial for maintaining the cell stemness of hAECs.^[^ ^26^ ^]^ We speculated that in vitro culture, the proliferation of hAECs may also be related to EMP properties, which was confirmed by our scRNA‐seq analysis and experimental results. Mature term hAECs underwent EMT during in vitro culture, entered a pEMT state similar to that in the early stage of development, acquired EMP properties, increased the expression of proliferation‐related genes, entered the cell cycle, and achieved in vitro expansion (Figure 2). However, with the continued advancement of the EMT process, an overabundance of mesenchymal properties leads to the aging of hAECs, making further proliferation difficult (Figure 3).

### 3.3 The Biological Function of theTGF‐β Signal Pathway on the Expansion of hAECs

The transforming growth factor‐beta (TGF‐β) signaling pathway plays an important role in regulating cell growth, differentiation, and development.^[^ ^27^ ^]^ By performing a gene set enrichment analysis (GSEA) of differentially expressed genes in proliferating cells, we found that the TGF‐β induced EMT pathway was upregulated, implying that the EMT process occurred during the proliferation of hAECs was likely induced by TGF‐β (Figure 5A). This was consistent with previous work that indicated the TGF‐β signaling pathway is one of the main pathways inducing EMT,^[^ ^28^ ^]^ and autocrine TGF‐β induces EMT in hAECs.^[^ ^16^ ^]^

SB431542 is an inhibitor of TGF‐β receptor kinase, which can inhibit the activities of ALK4, ALK5, and ALK7 and inhibit the process of EMT.^[^ ^29^ ^]^ In addition, research by Justin K. Ichida confirms that SB431542 induces the production of the transcription factor Nanog by inhibiting the TGF‐β signaling pathway and can replace SOX2 during the reprogramming of fibroblasts to iPSCs.^[^ ^30^ ^]^ SB431542 can also mitigate lupus nephritis by regulating B cells through the TLR9 / TGF‐β1 / PDGFB signaling pathway,^[^ ^31^ ^]^ restore mitochondrial homeostasis in retinal pigment epithelial cells by partially inhibiting high‐glucose‐induced EMT,^[^ ^29^ ^]^ and better maintain their epithelial characteristics by suppressing the EMT process in hAECs.^[^ ^16^ ^]^ Therefore, we hypothesized that the addition of SB431542 during the culture process may help maintain the pEMT state of hAECs by affecting EMT, thereby promoting cell proliferation and increasing cell yield.

To better monitor the changes in hAECs during in vitro culture, we screened for biomarkers that are closely related to the changes in EMP properties, including the epithelial markers CD326 and CD9 and the mesenchymal markers CD90 and vimentin (Figure 4). Furthermore, our SB431542 addition experiment revealed that SB431542 inhibited the EMT process in hAECs, maintained the expression of epithelial markers, inhibited the expression of mesenchymal markers, and better maintained the cobblestone‐shaped morphology. As hAECs underwent EMT and acquired EMP properties and proliferative capacity during passage P0, the addition of SB431542 at passage P0 inhibited the proliferation of hAECs but effectively promoted their proliferation and better maintained their epithelial characteristics at passage P1 (Figure 5). This is consistent with the results of Miki's team in 2022, where SB431542 was used to inhibit EMT in hAECs, thereby maintaining their stemness.^[^ ^26^ ^]^

Furthermore, by combining the addition of SB431542 with low‐density seeding (2 × 10^4^ cm^−2^), we optimized the culture scheme for hAECs and significantly increased the yield of P1 hAECs while preserving their karyotype (Figure S9, Supporting Information). Owing to their large surface‐to‐volume ratio, microcarriers can reduce the cell culture space, operational steps, contamination risk, and costs. When combined with a stirring system, they create a homogeneous cell culture environment conducive to easy scale‐up, allowing continuous monitoring and control of culture parameters such as pH, temperature, concentrations of CO_2_, nutrients, and waste. Moreover, since type I collagen constitutes the primary component of the basement membrane in the amniotic membrane,^[^ ^32^ ^]^ and our scRNA‐seq analysis indicated a potential proliferation‐enhancing effect on hAECs, we investigated the 3D cultivation of hAECs on CT3 microcarriers coated with a thin layer of acid‐denatured type I collagen using positively charged CT1 microcarriers as controls. As expected, the CT3 microcarriers were more conducive to the proliferation of hAECs while maintaining their epithelial properties (Figure S11, Supporting Information). The addition of SB431542 further enhanced the maintenance of EMP properties, proliferation, and cell viability, and reduced cellular senescence. Our results demonstrate that the combination of CT3 microcarriers with SB431542 enables the scale‐up of hAECs. (Figures 6 and 7).

### 3.4 Anti‐Inflammatory Effect of hAECs and their Application in Disease Treatment

hAECs secrete various immunoregulatory factors, including non‐classical major histocompatibility complex HLA‐G, interleukin‐10 (IL‐10), prostaglandin E2 (PGE2), and indoleamine 2,3‐dioxygenase (IDO). Through these immunoregulatory factors, hAECs exhibit their properties of suppressing the proliferation and activation of innate and adaptive immune cells.^[^ ^33^ ^]^ PGE2, a lipid signaling molecule associated with pain and inflammation, can protect organs against inflammation, oxidative stress, or fibrosis damage after injury and promote tissue repair and regeneration.^[^ ^34^ ^]^ In mesenchymal stem cells (MSCs) research, PGE2 is a recognized marker for the immunomodulatory ability of MSCs.^[^ ^35^ ^]^ For macrophages, PGE2 promotes their polarization toward the anti‐inflammatory M2 phenotype,^[^ ^36^ ^]^ whereas for T cells, PGE2 functions in a “concentration‐dependent” manner: at high concentrations, PGE2 suppresses the inflammatory response by inhibiting T helper 1 (Th1) subgroups, while simultaneously activating T helper 2 (Th2) subgroups; at low concentrations, PGE2 induces Th1 differentiation and promotes the inflammatory response through the PTGER4/EP4 signal.^[^ ^37^ ^]^ hAECs secrete PGE2, especially under the stimulation of proinflammatory factors; this secretion increases significantly, enhancing their immunomodulatory capabilities.^[^ ^33^ ^]^

We evaluated the immunomodulatory capabilities of hAECs by assessing their ability to secrete PGE2 and inhibit the proliferation of peripheral blood mononuclear cells (PBMCs) proliferation. The results showed that after optimization of the culture system, the ability of hAECs to secrete PGE2 and inhibit PBMC proliferation did not change (Figure 7; Figure S9, Supporting Information), proving the success of our culture system optimization.

## 4 Conclusion

Owing to their low immunogenicity and excellent immunoregulatory ability, hAECs are promising candidates for cell therapy. However, because of their limited proliferative capacity, the difficulty of achieving a scaled‐up culture restricts their application. In this study, combined with scRNA‐seq analysis and experimental validation, we report for the first time that the proliferative ability of hAECs during development and in vitro culture was preserved by EMP. By maintaining the EMP properties of hAECs with SB431542, we promoted their proliferation, providing a new approach for the proliferation culture of hAECs, realized the 3D cultivation of hAECs on denatured type I collagen‐modified microcarriers, and made scale‐up cultivation of hAECs possible.

## 5 Experimental Section

### Isolation and Expansion of hAECs

Human amniotic membranes were obtained from Shanghai East Hospital Affiliated with Tongji University and The International Peace Maternity & Child Health Hospital of China Welfare Institute. The use of human tissue was done with the approval of the Ethics Committee and the informed consent of the donors of Shanghai East Hospital (Ethics Approval Number: 2021‐TLSD‐011) and The International Peace Maternity & Child Health Hospital of China Welfare Institute (Ethics Approval Number: GLW‐2014‐11).

The isolation and cultivation methods of hAECs were similar to previous work,^[^ ^38^ ^]^ with few modifications. Briefly, human amniotic membranes obtained from healthy mothers undergoing cesarean section were first washed in PBS without calcium and magnesium to remove the blood clots and the extra connective tissue. Then, amniotic membranes were washed with PBS again and pre‐digested with pre‐digestion buffer (0.025% trypsin) for 10 min at 37 °C in a water bath to remove the residual blood cells. Then the amniotic membranes were digested with 0.25% trypsin for 30 min at 37 °C in a water bath, after which the digestion was terminated by adding the digestive termination medium. Passed the cell solution through a 50‐mesh cell filter, and centrifuged 5 min at 650g to collect hAECs. Cells were then cultured at a density of 8 × 10^4^ cells per cm^2^ in our MQ1 culture medium (F12/DMEM, Serum Replacement, L‐glutamine, nonessential amino acid, sodium pyruvate, and EGF). After the first passage, cells were cultured in our MQ2 culture medium (MQ1 added with Hydrocortisone). During the cultivation process, as a routine check, mycoplasma testing was conducted on each batch of PreP0 hAECs using the mycoplasma test kit (Sartorius, Germany).

To examine the influence of small‐molecule compounds SB431542, the culture density of hAECs, and the microcarriers on the proliferation of hAECs, hAECs were cultured in the medium with different concentrations of SB431542 or cultured at the density of 2, 4, 8 × 10^4^ cells per cm^2^ respectively, the expanding fold and the expression of different markers were examined to assess the effect of SB431542, the culture density and the macrocarriers on hAECs.

### scRNA‐Seq Data Analysis—Single Cell RNA‐Seq Data Process and Further Analysis

For single‐cell RNA‐sequencing (scRNA‐seq), hAECs collected from human amniotic membranes (PreP0) of different gestational ages or cultured P0‐hAECs, P1‐hAECs, and P2‐hAECs were resuspended in the culture media and transported to OE Biotech Co., Ltd. (Shanghai, China) for sequencing. The single‐cell RNA sequencing (scRNA‐seq) data was analyzed and integrated using Seurat R package (v.4.3.0.1, https://github.com/satijalab/seurat).^[^ ^39^, ^40^ ^]^ Following this, the dataset underwent meticulous filtration based on the total number of identified genes, unique molecular identifiers (UMI), and the proportion of mitochondrial genes. Subsequently, the raw counts were normalized (using NormalizeData) to determine gene expression levels relative to the total counts and then subjected to data scaling (using ScaleData). Principal component analysis (PCA; using RunPCA) was then applied to the refined dataset. To integrate various scRNA‐seq datasets, each dataset's count matrices were individually filtered, normalized, and then harmonized using Harmony (v.1.0.0, https://github.com/immunogenomics/harmony).^[^ ^41^ ^]^ Subsequently, the data was embedded into a low‐dimensional space via unified manifold approximation and projection (UMAP; using RunUMAP). Finally, cell clusters were generated using the FindClusters function in the Seurat R package.

In further analysis, cell cycle scoring was performed on all cells to determine the phase of the cell cycle each cell was in. To gain deeper insights into cell types, human cell data (HumanPrimaryCellAtlasData) from the cell dex package was referenced, and SingleR was employed for cell annotation, covering annotations for subgroups, samples, and all cells. Following the filtering of unwanted cells based on combined annotation and marker expression, Monocle 2 (v.2.22.0; reduction_method = “DDRTree”, residualModelFormulaStr = “∼num_genes_expressed”) and Monocle 3 (v3.1.0.0; resolution = 1e‐3) were utilized for pseudotime analysis of the remaining epithelial cells.

### scRNA‐Seq Data Analysis—Single Cells WGCNA

Due to the pronounced sparsity of single‐cell data and the high sensitivity of weighted gene co‐expression network analysis (WGCNA)^[^ ^42^, ^43^, ^44^, ^45^ ^]^ to data sparsity, the construction of metacells to replace the original expression matrix becomes necessary. Employing the k‐Nearest Neighbors (KNN) algorithm, small groups of similar cells originating from the same biological source were identified, and their average expression was calculated to generate the metacell gene expression matrix. Subsequently, the WGCNA R package (v.1.71; http://www.genetics.ucla.edu/labs/horvath/CoexpressionNetwork/Rpackages/WGCNA)^[^ ^42^ ^]^ was loaded to conduct co‐expression network analysis on the new expression matrix. Initially, power calculation was conducted on Pearson correlation values (using pickSoftThreshold) to determine the appropriate soft threshold, with the selection of a power value above 0.9 for a robust scale‐free topology model fit. Once the soft threshold was determined, a network was built (using blockwise modules) to identify distinct modules of key genes.

### scRNA‐Seq Data Analysis—Differential Gene Expression and Gene Sets Enrichment Analyses

The differential expression genes (DEGs) among distinct clusters, samples, or cell types were identified using the FindMarkers function in the Seurat R package (v.4.3.0.1, https://github.com/satijalab/seurat),^[^ ^39^, ^40^ ^]^ with a minimum log‐fold change of 0.25. Unless explicitly stated otherwise, the gene expression data presented in violin plots, feature plots, and dot plots throughout this study were derived from computations based on the “data” and “scaled data” assays within the Seurat object. Following the identification of DEGs, gene set enrichment analysis was performed using pathway gene sets obtained from GO, KEGG, and GSEA databases, with the human genome annotation package org.Hs.eg.db (v.3.14.0)^[^ ^46^ ^]^ consulted for gene annotation references. Enrichment analyses for GO and KEGG pathways were executed using the enrichGO and enrichKEGG functions from the clusterProfiler package (v.4.2.2; https://github.com/YuLab‐SMU/clusterProfiler),^[^ ^47^ ^]^ respectively, and visualized using barplot and dotplot functions from the ggplot2 package.^[^ ^48^ ^]^ GSEA enrichment analysis of DEGs was implemented via the GSEA function from the GSEABase package (v.1.56.0; https://github.com/Bioconductor/GSEABase),^[^ ^49^ ^]^ leveraging pathway gene sets sourced from MigDB. The visualization of GSEA results was facilitated through the gseaplot2 function from the enrichplot package (v.1.14.2; https://yulab‐smu.top/biomedical‐knowledge‐mining‐book)^[^ ^50^ ^]^ and the ggplot function from the ggplot2 package.

### qPCR

Cells were lysed and total RNA was extracted using TaKaRa MiniBEST Universal RNA Extraction Kit (TaKaRa, Japan), and 500 ng RNA was used to generate cDNAs using PrimeScript RT Master Mix (TaKaRa, Japan) by PROFLEX 3 × 32‐WELL PCR system (Thermo, USA) according to the manufacture's instructions. qPCR was performed using TB Green Premix Ex Taq II (TaKaRa, Japan) by QuantStudio 5 Real Time PCR System (Thermo, USA). The gene expression levels were normalized against endogenous  β‐actin (ACTB). Specific primer pairs are shown in Table S5 (Supporting Information) and the specificity of the qPCR primers was verified with negative and positive control cells (Figure S12A, Supporting Information).

### Flow Cytometry Analysis

Collected hAECs were first washed with PBS, and then incubated in PBS with 1% bovine serum albumin (BSA) for 10 min to prevent non‐specific adsorption. After that, the antibodies of surface markers were added and incubated for 30 min at 4 °C followed by washing with PBS and examination. For intracellular markers, hAECs were first fixed with 4% paraformaldehyde for 10 min at 4 °C and permeabilized with 0.2% Triton X‐100 for 4 min at 4 °C, after which hAECs were incubated with 1% BSA for 10 min at room temperature, followed by the intracellular antibodies’ incubation for 30 min at 4 °C. Single‐staining method was used for all antibodies. The samples were tested by CytoFLEX Flow Cytometer (BECKMAN COULTER Life Sciences, USA) and the data was analyzed with FlowJo software ver. 10.8.1. In our study, the single‐staining method made the gating strategy relatively straightforward during analysis. Cells were first gated by SSC‐H versus FSC‐H, then the positive populations of the antibodies were plotted against SSC‐H using cells processed with relative isotype antibodies as negative controls. The antibodies used for flow cytometry analysis are listed in Table S6 (Supporting Information). The specificity of all antibodies was verified with recommended cell lines as negative and positive controls (Figure S12B, Supporting Information).

### Cell Proliferation Assay with Cell Counting Kit‐8 (CCK8)

To test the proliferation of hAECs, cells in 96‐well plates treated with different concentrations of SB431542 were tested according to the manufacturer's instruction of the CCK‐8 kit (Beyotime, China). The absorbance at 450 nm was examined by Spark Multimode Microplate Reader (Tecan, Switzerland).

### Immunocytochemistry

Cells cultured on cover glasses were fixed in 4% paraformaldehyde for 20 min and permeabilized with 0.2% Triton X‐100 for 10 min respectively at room temperature. Then the samples were incubated with blocking buffer (5% BSA) for 1 h at room temperature followed by the incubation with primary antibodies over night at 4 °C and secondary antibodies and DAPI for 1 h at room temperature. After washing, samples were mounted and the images were taken with Olympus IX51 inverted fluorescence microscope (Olympus, Japan). The antibodies used here are listed in Table S6 (Supporting Information).

### SA‐β‐Gal Staining

For freshly isolated hAECs, cells, and hAECs‐MCs in culture, the senescence examination was performed according to the manufacturer's instruction of senescence‐associated β‐galactosidase kit (SA‐β‐Gal kit, Beyotime, China). To detect the senescence of hAECs in amniotic membrane, full‐term fetal amniotic membranes were collected and fixed with 4% paraformaldehyde, after dehydration, the amniotic membranes were then subjected to paraffin embedding. Paraffin‐embedded tissue sections (5 µm) were cut with HistoCore BIOCUT Mechanical Manual Rotary Microtome (Leica, Germany) and after dewaxing and rehydration, the tissue sections were used to check the senescence of hAECs in amniotic membranes. Images were captured with a TS2+DS inverted microscope (Nikon, Japan).

### Cell Viability Measurement

Cell morphology and viability on microcarriers (MCs) were assessed using laser‐scanning confocal microscopy (CLSM, Nikon A1R, Japan). hAECs‐MCs were harvested in centrifuge tubes, washed twice with PBS, and stained with Calcein/PI Assay Kit (C2015L, Bytotime, China). Live cells were labeled with Calcein AM (green, Ex/Em = 494/517 nm), while dead cells were stained with propidium iodide (PI) (red, Ex/Em = 535/617 nm). Cell nuclei were counterstained with 4′, 6‐Diamidino‐2‐Phenylindole (DAPI, 62248, Thermo Scientific, USA) for 5 min. The excitation/emission wavelengths of DAPI were 341/452 nm.

### Scanning Electron Microscopy (SEM)

To examine the surface morphology of Cytodex 3 MCs and the interaction of hAECs and Cytodex 3 MCs, Cytodex 3 MCs, and dry hAECs‐MCs, obtained after gradient ethanol dehydration using 50%, 60%, 70%, 80%, 90%, and 100%, were evaluated by scanning electron microscopy (SEM, JEOL, Japan).

### RNA‐Seq

For RNA‐sequencing (RNA‐seq), Total RNA was extracted using the TRIzol reagent (Invitrogen, CA, USA) according to the manufacturer's protocol and then transported to OE Biotech Co., Ltd. (Shanghai, China) for sequencing and analysis. Raw mRNA sequencing was conducted on an Illumina NovaSeq6000 sequencer in PE150 sequencing mode. Clean reads were generated using Seqtk, followed by genome mapping with Hisat2 (version: 2.0.4) and alignment and gene read count quantification using Stringtie (version: 1.3.0). The genomic version used was GRCh38 (hg38).

### PGE2 Secretion Capacity Assay

To detect the prostaglandin E2 (PGE2) secretion capacity, hAECs were suspended in our analytical culture medium (F12/DMEM, Fetal Bovine Serum (FBS), L‐glutamine, nonessential amino acid, sodium pyruvate, and EGF) and adjusted to the concentration of 1 × 10^5^ cells mL^−1^. After that, 2 mL cellular suspension was added and cultured in the 12‐well cell culture plastics for 48 h. Then the culture medium was changed and cultured for a further 48 h. The cell culture supernate was collected and the concentration of PGE2 was tested with PGE2 ELISA Kit (cayman chemical, USA).

### PBMC Proliferation Suppression Assay

Peripheral blood mononuclear cells (PBMCs) were labeled with CFSE (CFSE, 2 µM) (Life Technologies) to track their proliferation and PBMCs were activated with 10 µg mL^−1^ phytohemagglutinin (PHA‐P) (Sigma). 2 × 10^5^ hAECs were seeded in 12 well‐plates in our analytical culture medium for 48 h. Then, activated PBMCs were added at the concentration of 1 × 10^6^ cells/well on hAECs in RPMI‐1640 complete culture medium. As a positive and negative control condition, activated or non‐activated PBMCs were cultured alone. After 5 days of co‐culture, PBMCs were harvested and the proliferation was quantified by flow cytometry.

### Karyotype Analysis

The cultured P1‐hAECs that reach 90% confluence were transported to the Company of Labway (Shanghai, China) for karyotype analysis. The karyotype analysis was performed according to the standard of pharmacopoeia.

### Statistical Analysis

Statistical analyses were performed using GraphPad prism 9.0 software. An unpaired two‐tailed student's t‐test was used for the comparison of two samples. For multiple samples comparison, one‐way ANOVA followed by a Tukey post hoc test was utilized. Additionally, two‐way ANOVA followed by a Sidak post hoc test was employed for repeated measures of two samples. In all experiments, the data sets were calculated and expressed as mean ± standard deviation (SD). The experiments were conducted at least three times, and p < 0.05 was considered statistically significant (*p* < 0.05 (^*^), *p* < 0.01 (^**^), and *p* < 0.001 (^***^)).

### Ethics Approval and Consent to Participate

Human amniotic membranes were obtained from Shanghai East Hospital Affiliated with Tongji University and The International Peace Maternity & Child Health Hospital of China Welfare Institute. The use of human tissue was done with the approval of the Ethics Committee and the informed consent of the donors of Shanghai East Hospital (Ethics Approval Number: 2021‐TLSD‐011) and The International Peace Maternity & Child Health Hospital of China Welfare Institute (Ethics Approval Number: GLW‐2014‐11).

## Conflict of Interest

The authors declare no conflict of interest.

## Supporting information



Supporting Information

Supplemental Video 1

Supporting Information

## Data Availability

The data that support the findings of this study are available on request from the corresponding author. The data are not publicly available due to privacy or ethical restrictions.
