# Supplementary material for: Scale‐Up of Human Amniotic Epithelial Cells Through Regulation of Epithelial‐Mesenchymal Plasticity Under Defined Conditions
Source: Adv Sci (Weinh). 2025 Jan 13;12(11):2408581. doi: 10.1002/advs.202408581 (PMC11923953; doi:10.1002/advs.202408581)
Supplement: Supplementary file 1 — Supporting Information [file ADVS-12-2408581-s003.pdf]

ADVANCED SCIENCE
Open Access

Supporting Information

for *Adv. Sci.*, DOI 10.1002/advs.202408581

Scale-Up of Human Amniotic Epithelial Cells Through Regulation of Epithelial-Mesenchymal Plasticity Under Defined Conditions

Wangping Hao, Yi Luo, Jia Tian, Yuefeng Lu, Yangyang Cui, Ying Zhang, Xiao Jin, Hongjuan Ye, Mengqi Lu, Jinjia Song, Weiqing Zhou, Wencheng Zhang* and Zhiying He**

Scale-up of Human Amniotic Epithelial Cells through Regulation of Epithelial-Mesenchymal Plasticity under Defined Conditions

Figure S1. The scoring results of the gene sets provided in Supplementary Material Genelist, and the SingleR scores of the samples along with the markers' expression ploted in UMAP.

A. Violin plot of the gene set (HALLMARK EPITHELIAL MESENCHYMAL TRANSITION, partial EMT, Epithelial, Mesenchymal in Supplementary Material **Genelist**) scores calculated by AddModuleScore function and shown in each cluster.

B. Display of cluster distribution in a scatter plot with Epithelial signature score and Mesenchymal signature score as horizontal and vertical axes, respectively.

C. SingleR score heat map for the PreP0 samples.

D. Display of strongly expressed epithelial (KRT18) and mesenchymal (TGFBI) markers and their co-expression in UMAP.

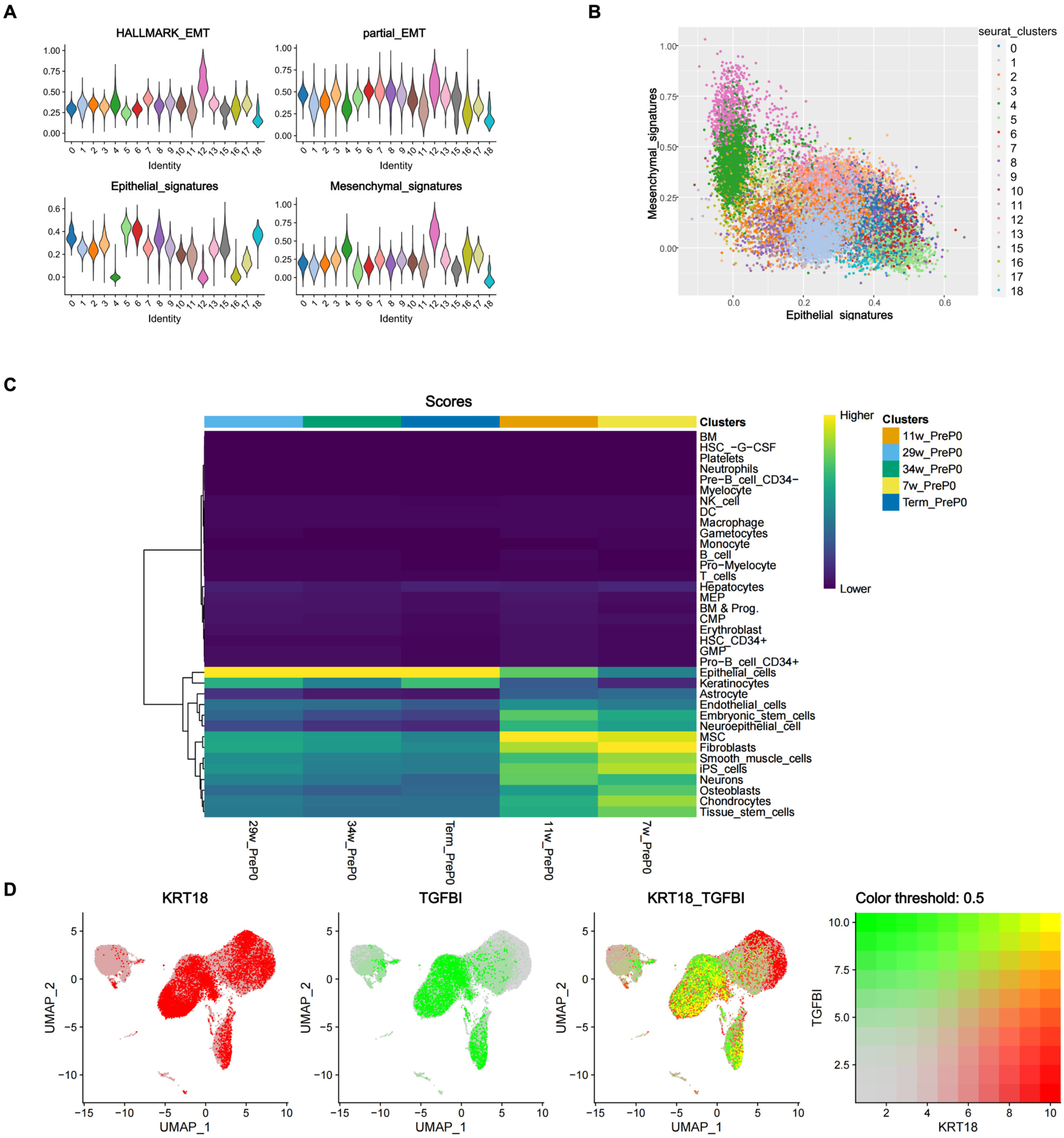
A
HALLMARK_EMT
partial_EMT
Epithelial_signatures
Mesenchymal_signatures
Identity
B
seurat_clusters
Mesenchymal_signatures
Epithelial_signatures
C
Scores
Clusters
Higher
Lower
11w_PreP0
29w_PreP0
34w_PreP0
7w_PreP0
Term_PreP0
BM
HSC_-G-CSF
Platelets
Neutrophils
Pre-B_cell_CD34-
Myelocyte
NK_cell
DC
Macrophage
Gametocytes
Monocyte
B_cell
Pro-Myelocyte
T_cells
Hepatocytes
MEP
BM & Prog.
CMP
Erythroblast
HSC_CD34+
GMP
Pro-B_cell_CD34+
Epithelial_cells
Keratinocytes
Astrocyte
Endothelial_cells
Embryonic_stem_cells
Neuroepithelial_cell
MSC
Fibroblasts
Smooth_muscle_cells
iPS_cells
Neurons
Osteoblasts
Chondrocytes
Tissue_stem_cells
D
KRT18
TGFBI
KRT18_TGFBI
Color threshold: 0.5
UMAP_1
UMAP_2

Figure S2. hAECs isolated from full-term amniotic membranes were mature amniotic epithelial cells.

A. Results of scRNA-seq analysis of the expression of mesenchymal markers Vimentin, CD90 (THY1), and CD105 (ENG), and epithelial markers CDH1 (CD324), KRT18 (CK18), and CD9 in freshly isolated full-term hAECs.

B. The expression of mesenchymal markers and epithelial markers in freshly isolated full-term hAECs were validated by qPCR detection with positive and negative control cells. Data were normalized with the positive control group set as 1 and presented as mean ± SD. one-way ANOVA was used for the comparison. $n \geq 3$， $p < 0.05$ (*), $p < 0.01$ (**), and $p < 0.001$ (***).

C. The flow cytometry analysis of the expression of epithelial and mesenchymal markers in freshly isolated full-term hAECs and their corresponding statistical results ($n \geq 3$).

D. The results of scRNA-seq analysis of the expression of pluripotency-related markers OCT4 (POU5F1), NANOG, SOX2 and telomerase enzyme TERT and TERC in freshly isolated full-term hAECs.

E. The expression of pluripotency markers OCT4, NANOG, SOX2 in freshly isolated full-term hAECs were validated by qPCR detection with positive and negative control cells. Data were normalized with the positive control group set as 1 and presented as mean ± SD. one-way ANOVA was used for the comparison. $n \geq 3$， $p < 0.05$ (*), $p < 0.01$ (**), and $p < 0.001$ (***).

F. The expression of pluripotency markers OCT4, NANOG, SOX2 in freshly isolated full-term hAECs were validated by immunofluorescence detection with iPSCs and HFL1 as positive and negative control cells.

G. Flow cytometry analysis of the expression of pluripotency markers (OCT4, NANOG, SOX2, TRA1-60, TRA1-81, SSEA3, SSEA4) in freshly isolated full-term hAECs.

H. Statistical results of the expression of pluripotency markers examined by flow cytometry analysis ($n \geq 3$).

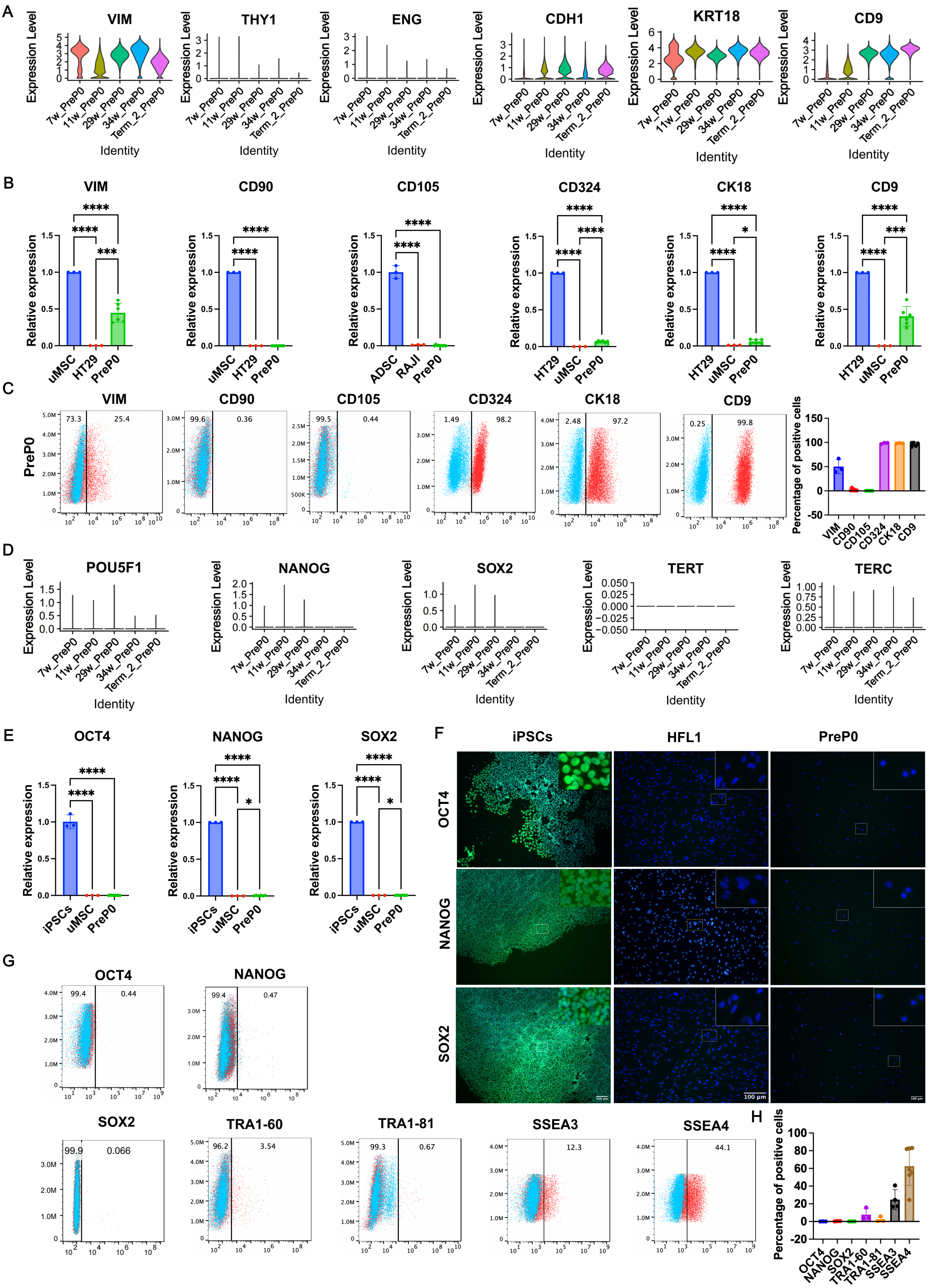
A
VIM
THY1
ENG
CDH1
KRT18
CD9
Expression Level
7w_PreP0
11w_PreP0
29w_PreP0
34w_PreP0
Term_2_PreP0
Identity
B
VIM
CD90
CD105
CD324
CK18
CD9
Relative expression
uMSC
HT29
PreP0
ADSC
RAJI
C
PreP0
Percentage of positive cells
D
POU5F1
NANOG
SOX2
TERT
TERC
E
OCT4
NANOG
SOX2
iPSCs
F
iPSCs
HFL1
PreP0
OCT4
NANOG
SOX2
100 μm
G
OCT4
NANOG
SOX2
TRA1-60
TRA1-81
SSEA3
SSEA4
H
Percentage of positive cells

Figure S3. A. Eight key gene modules obtained using WGCNA.

B. Eight key gene modules were obtained using WGCNA. Each cell was scored by AddModuleScore using the gene set of each module and the scores were visualized by FeaturePlot.

C. GO enrichment analysis of 6 modules and the top 20 terms were visualized with barplots.

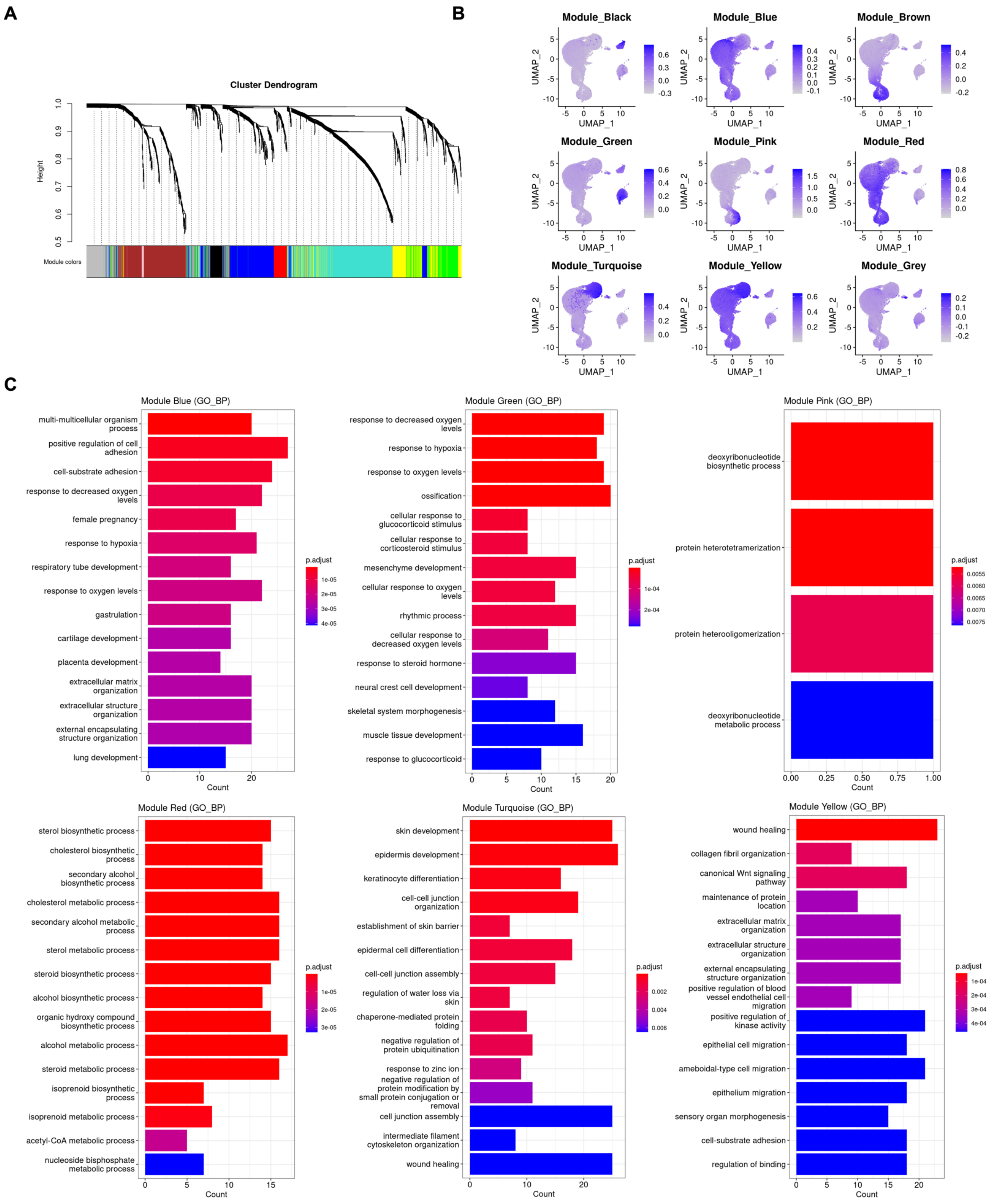
A
Cluster Dendrogram
Height
Module colors
B
Module_Black
Module_Blue
Module_Brown
Module_Green
Module_Pink
Module_Red
Module_Turquoise
Module_Yellow
Module_Grey
UMAP_1
UMAP_2
C
Module Blue (GO_BP)
multi-multicellular organism process
positive regulation of cell adhesion
cell-substrate adhesion
response to decreased oxygen levels
female pregnancy
response to hypoxia
respiratory tube development
response to oxygen levels
gastrulation
cartilage development
placenta development
extracellular matrix organization
extracellular structure organization
external encapsulating structure organization
lung development
Count
p.adjust
Module Green (GO_BP)
response to decreased oxygen levels
response to hypoxia
response to oxygen levels
ossification
cellular response to glucocorticoid stimulus
cellular response to corticosteroid stimulus
mesenchyme development
cellular response to oxygen levels
rhythmic process
cellular response to decreased oxygen levels
response to steroid hormone
neural crest cell development
skeletal system morphogenesis
muscle tissue development
response to glucocorticoid
Count
p.adjust
Module Pink (GO_BP)
deoxyribonucleotide biosynthetic process
protein heterotetramerization
protein heterooligomerization
deoxyribonucleotide metabolic process
Count
p.adjust
Module Red (GO_BP)
sterol biosynthetic process
cholesterol biosynthetic process
secondary alcohol biosynthetic process
cholesterol metabolic process
secondary alcohol metabolic process
sterol metabolic process
steroid biosynthetic process
alcohol biosynthetic process
organic hydroxy compound biosynthetic process
alcohol metabolic process
steroid metabolic process
isoprenoid biosynthetic process
isoprenoid metabolic process
acetyl-CoA metabolic process
nucleoside bisphosphate metabolic process
Count
p.adjust
Module Turquoise (GO_BP)
skin development
epidermis development
keratinocyte differentiation
cell-cell junction organization
establishment of skin barrier
epidermal cell differentiation
cell-cell junction assembly
regulation of water loss via skin
chaperone-mediated protein folding
negative regulation of protein ubiquitination
response to zinc ion
negative regulation of protein modification by small protein conjugation or removal
cell junction assembly
intermediate filament cytoskeleton organization
wound healing
Count
p.adjust
Module Yellow (GO_BP)
wound healing
collagen fibril organization
canonical Wnt signaling pathway
maintenance of protein location
extracellular matrix organization
extracellular structure organization
external encapsulating structure organization
positive regulation of blood vessel endothelial cell migration
positive regulation of kinase activity
epithelial cell migration
ameboidal-type cell migration
epithelium migration
sensory organ morphogenesis
cell-substrate adhesion
regulation of binding
Count
p.adjust

Figure S4. A. Heatmap showing the expression of proliferation and senescence markers across different subgroups.

B. UMAP plot of cell cycle scoring results for all cells.

C. Heatmap displaying the results of cell annotation for cell subgroups using SingleR.

D. Scatter plot showing the connection between E/M correlation scores and cell pEMT states.

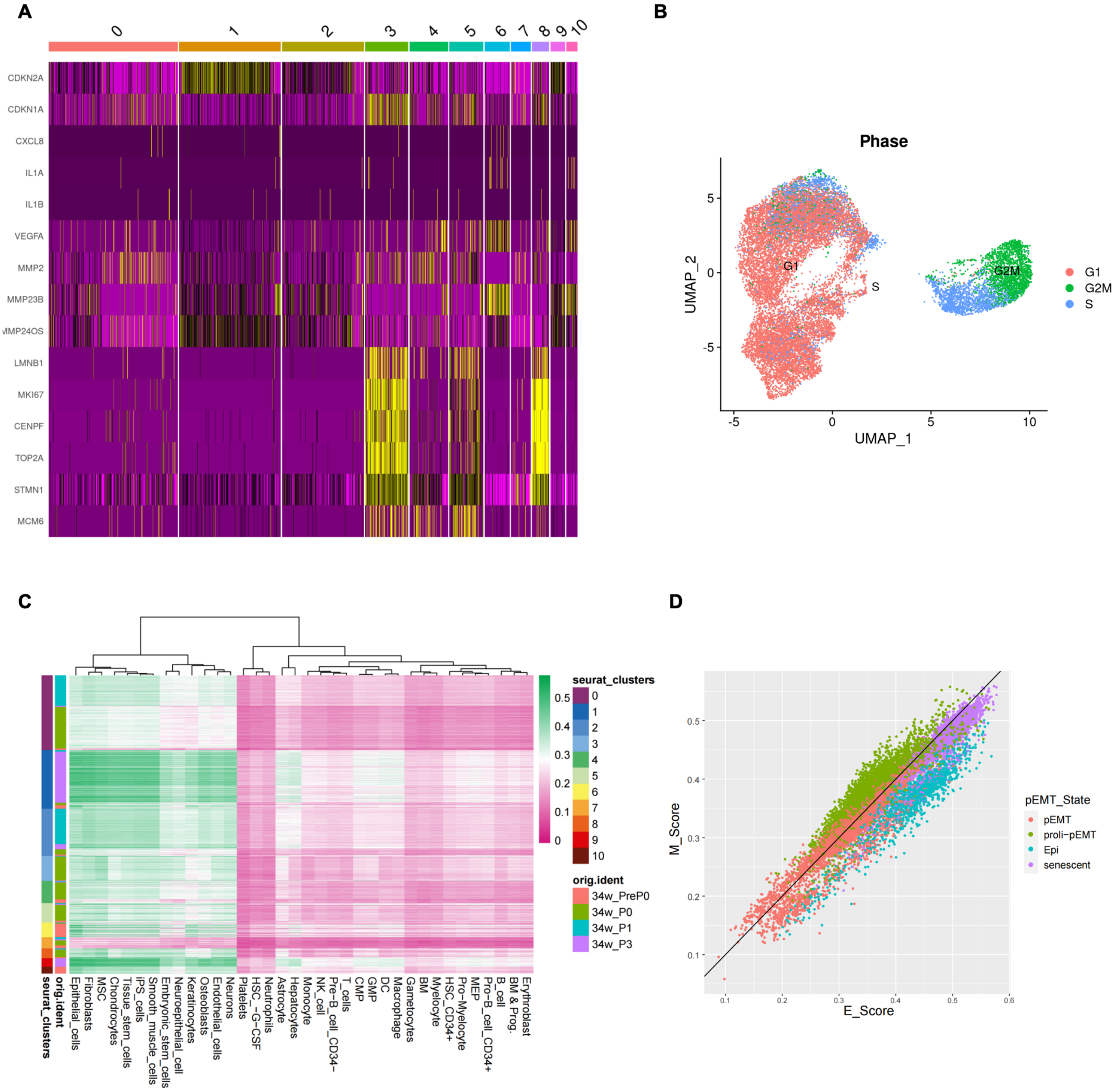

Figure S5. Integration and annotation results for 18 samples and epithelial-mesenchymal scoring for each subgroup.

A. Integration results and cell annotation for the 18 samples.

B. The epithelial and mesenchymal scores of different subpopulations obtained by SingleR and the corresponding E/M ratio were visualized by violin plots.

C. Violin plots depicting the epithelial and mesenchymal scoring results of different subgroups.

D. Violin plots illustrating the epithelial and mesenchymal scoring results based on cell type grouping.

E. Violin plot displaying the E/M ratio based on cell type grouping.

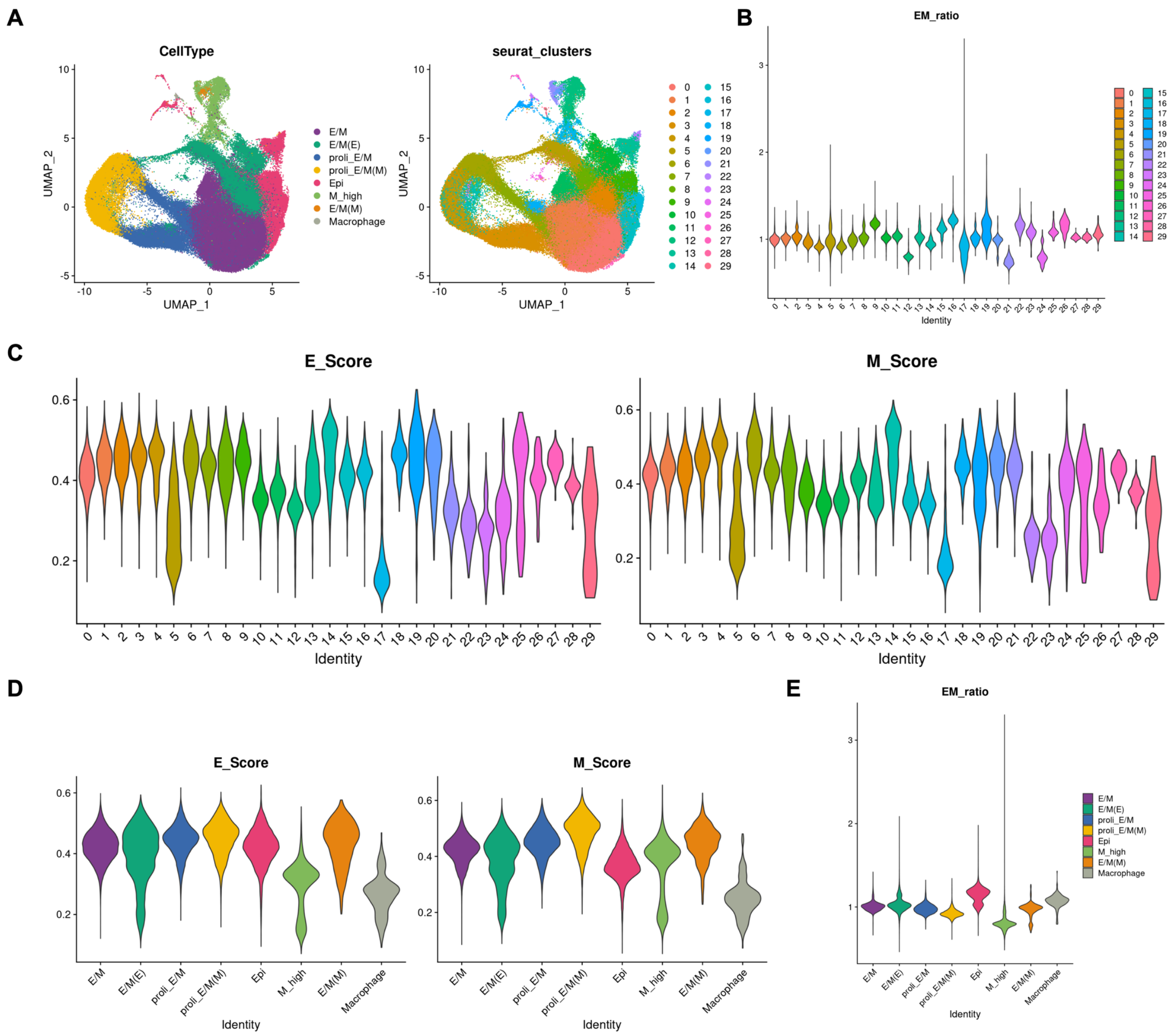

Figure S6. SB431542 inhibited the proliferation of passage P0 hAECs.

A. Effects of SB431542 on cell adhesion and morphology of P0 hAECs. Bar=50 μm

B. Impact of adding SB431542 on the expression of epithelial marker CD326.

C. CCK8 assay to assess the effects of adding 2.5, 5, 10 μM SB431542 from passage P0 on the proliferation of passages P0 and P1 hAECs. Data were presented as mean ± SD, and one-way ANOVA was used for the comparison. n ≥3， $p < 0.05$ (*), $p < 0.01$ (**), and $p < 0.001$ (***).

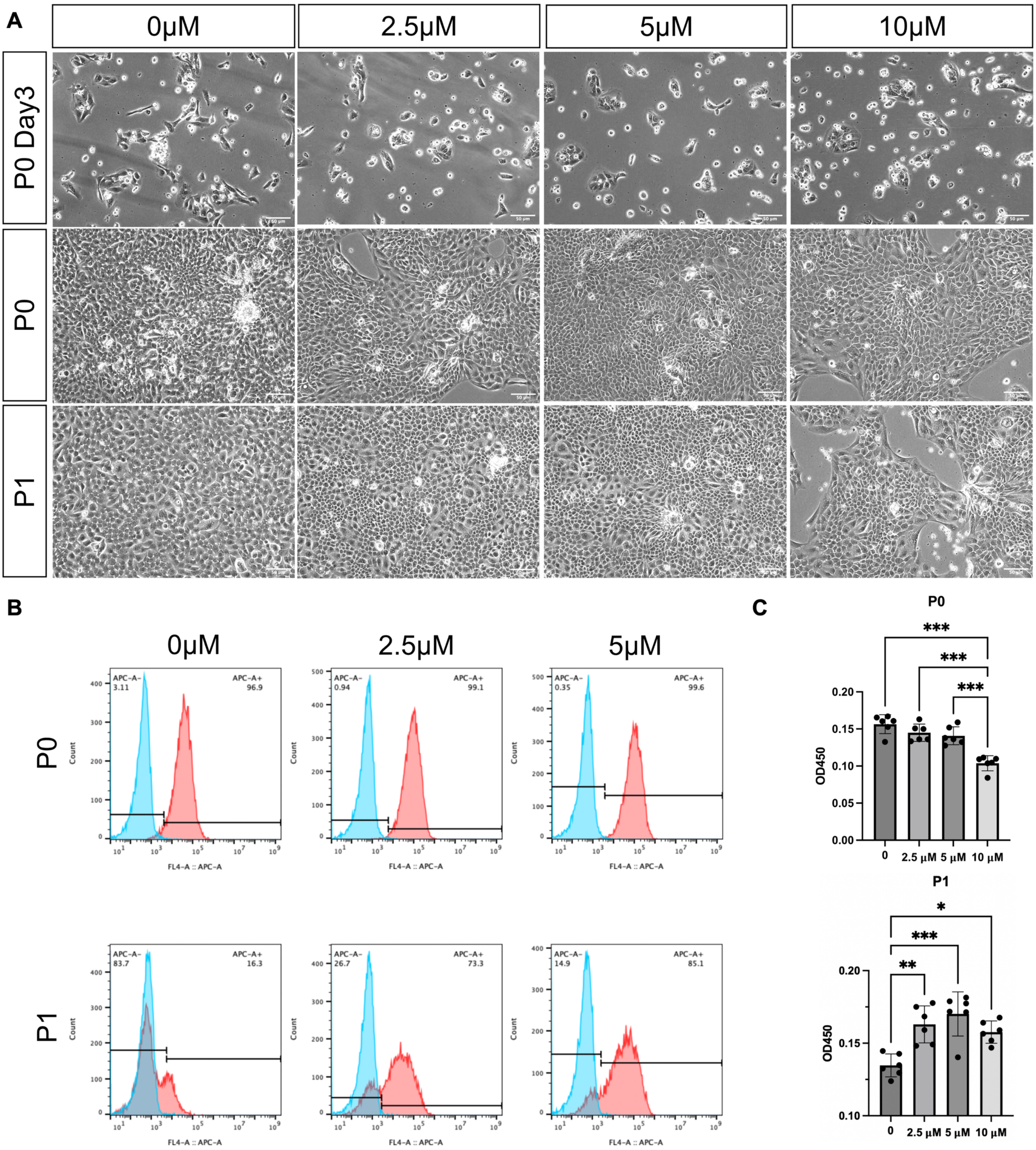

Figure S7. There was a primary inhibitory effect of SB431542 on the proliferation of P0 hAECs, while a promotion effect on P1 and P2 hAECs when the addition of SB431542 starting from passage P0.

A. Effects of adding SB431542 starting from passage P0 on the cell morphology of passages P0, P1, and P2 hAECs. Bar=50 μm

B. CCK8 assay to assess the impact of adding SB431542 on the proliferation of passages P0, P1, and P2 hAECs. Data were presented as mean ± SD, and one-way ANOVA was used for the comparison. n ≥3， $p < 0.05$ (*), $p < 0.01$ (**), and $p < 0.001$ (***).

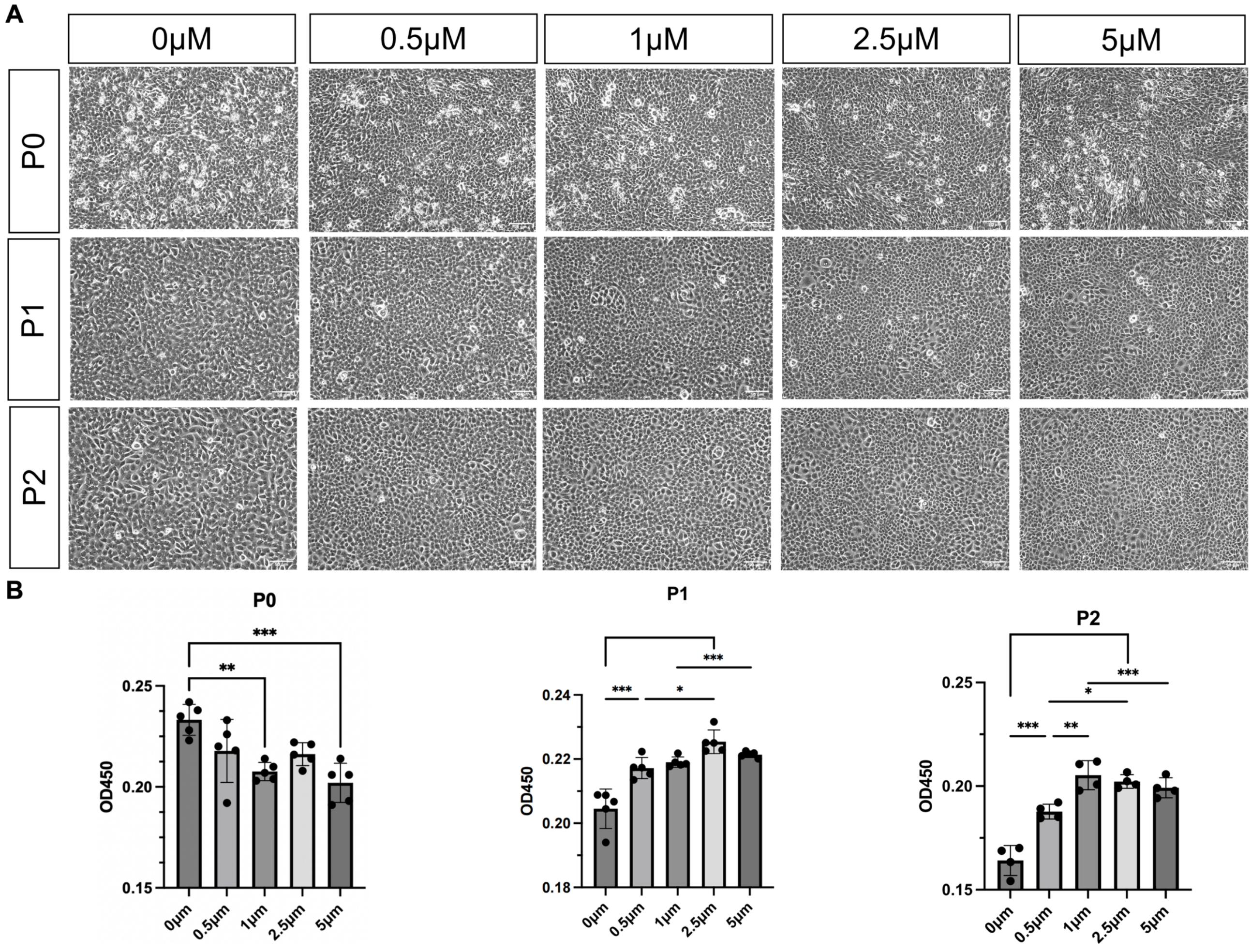

Figure S8. The addition of SB431542 from passage P1 promoted the proliferation of hAECs by maintaining the pEMT state.

A. Effects of SB431542 addition on the cell morphology of hAECs from passages P1, P2, P3, and P4. Bar=50 μm

B. CCK8 assay to evaluate the impact of adding SB431542 on the proliferation of passages P1, P2, P3, and P4 hAECs. Data were presented as mean ± SD, and one-way ANOVA was used for the comparison. $n \geq 3$， $p < 0.05$ (*), $p < 0.01$ (**), and $p < 0.001$ (***).

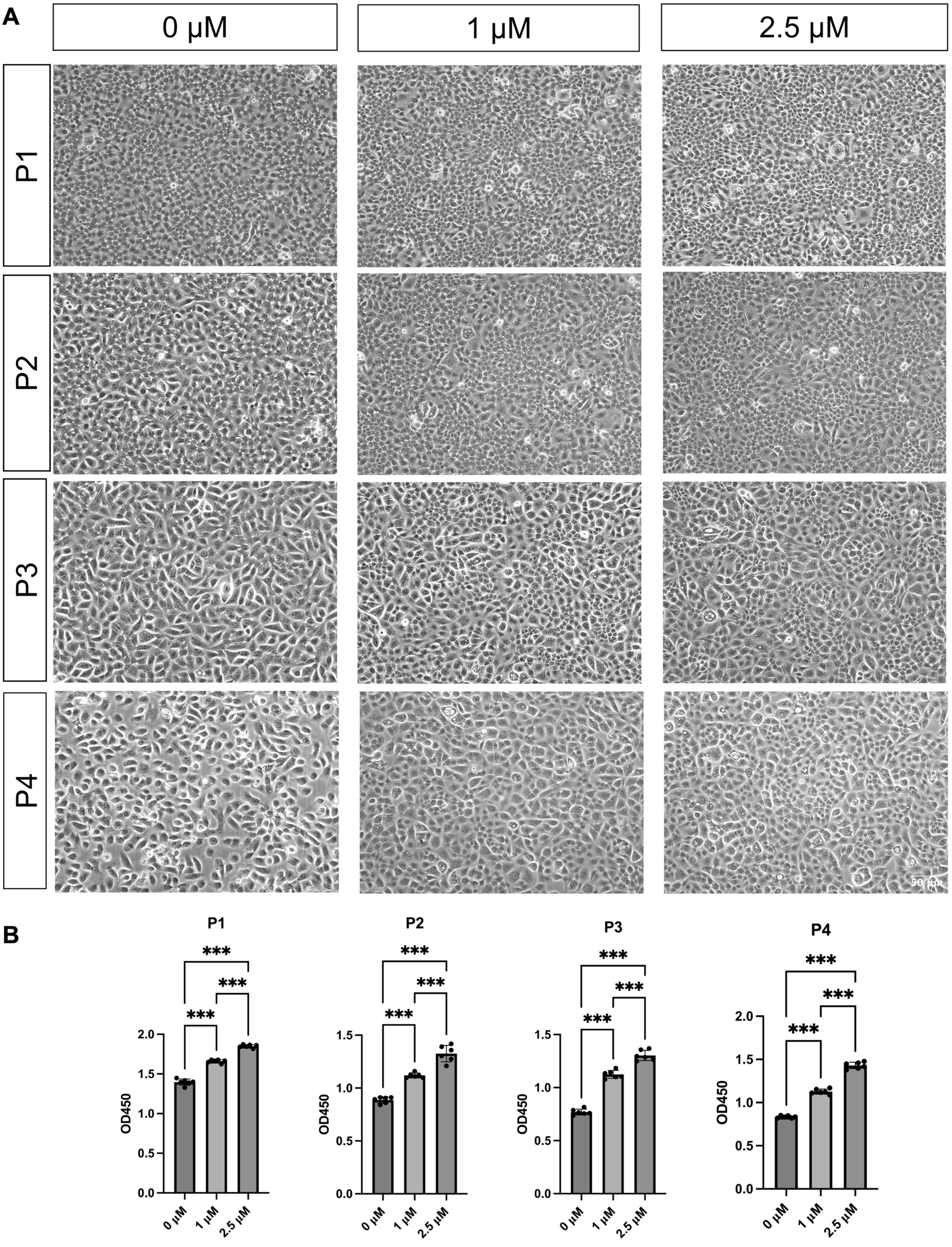
A
0 µM
1 µM
2.5 µM
P1
P2
P3
P4
B
P1
P2
P3
P4
***
OD450
0 µM
1 µM
2.5 µM

Figure S9. Optimization of the culture protocol for hAECs based on the addition of SB431542, while maintaining their functionality.

A. Morphology of passages P0 and P1 hAECs under seeding densities of 2, 4, 8 × $10^4/cm^2$. Bar=50 μm.

B. Expression levels of epithelial markers CD9, CD326, and mesenchymal markers Vimentin, CD90 in passage P1 hAECs under seeding densities of 2, 4, 8 × $10^4/cm^2$ were detected by flow cytometry.

C. The improvement of cell proliferation of hAECs under seeding densities of 4 × $10^4/cm^2$, 2 × $10^4/cm^2$ compared to 8 × $10^4/cm^2$ (Data were normalized with the group of 8 × $10^4/cm^2$ set as 1), along with statistical analysis results from Figure S8B. Data were presented as mean ± SD, one-way ANOVA and unpaired two-tailed student's t-test were used for the comparison. n ≥3， $p < 0.05$ (*), $p < 0.01$ (**), and $p < 0.001$ (***).

D. Effect of adding 1 μM SB431542 on the morphology of passage P1 hAECs at a seeding density of 2×$10^4/cm^2$. Bar=100 μm

E. Flow cytometry analysis was used to examined the expression of epithelial markers CD9, CD326, and mesenchymal markers Vimentin, CD90 in passage P1 hAECs under seeding density of 2 × $10^4/cm^2$ with the addition of SB431542.

F. The improvement of cell proliferation of hAECs under seeding densitie of 2 × $10^4/cm^2$ with the addition of 1 μM SB431542 compared to the control group without SB431542 (Data were normalized with the control group), along with statistical analysis results from Figure 8E. Data were presented as mean ± SD, and unpaired two-tailed student's t-test was used for the comparison. n ≥3， $p < 0.05$ (*), $p < 0.01$ (**), and $p < 0.001$ (***).

G. Incucyte analysis of the impact of adding 1 μM SB431542 on the proliferation of passage P1 hAECs under a seeding density of 2 × $10^4/cm^2$.

H. RNA-seq analysis was used to assess the correlation of hAECs between P1 hAECs cultured under seeding density 8 × $10^4/cm^2$ and under seeding density 2 × $10^4/cm^2$ with the addition of 1 μM SB431542.

I, J. PGE2 secretion and PBMC proliferation suppression assay in passage P1 hAECs under seeding density 8 × $10^4/cm^2$ and seeding density 2 × $10^4/cm^2$ with the addition of 1 μM SB431542. Data were presented as mean ± SD, and unpaired two-tailed student's t-test was used for the comparison. n ≥3， $p < 0.05$ (*), $p < 0.01$ (**), and $p < 0.001$ (***).

K. Karyotype analysis result of passage P1 hAECs cultured under seeding density of 2 × 10^4/cm^2 with the addition of 1 μM SB431542.

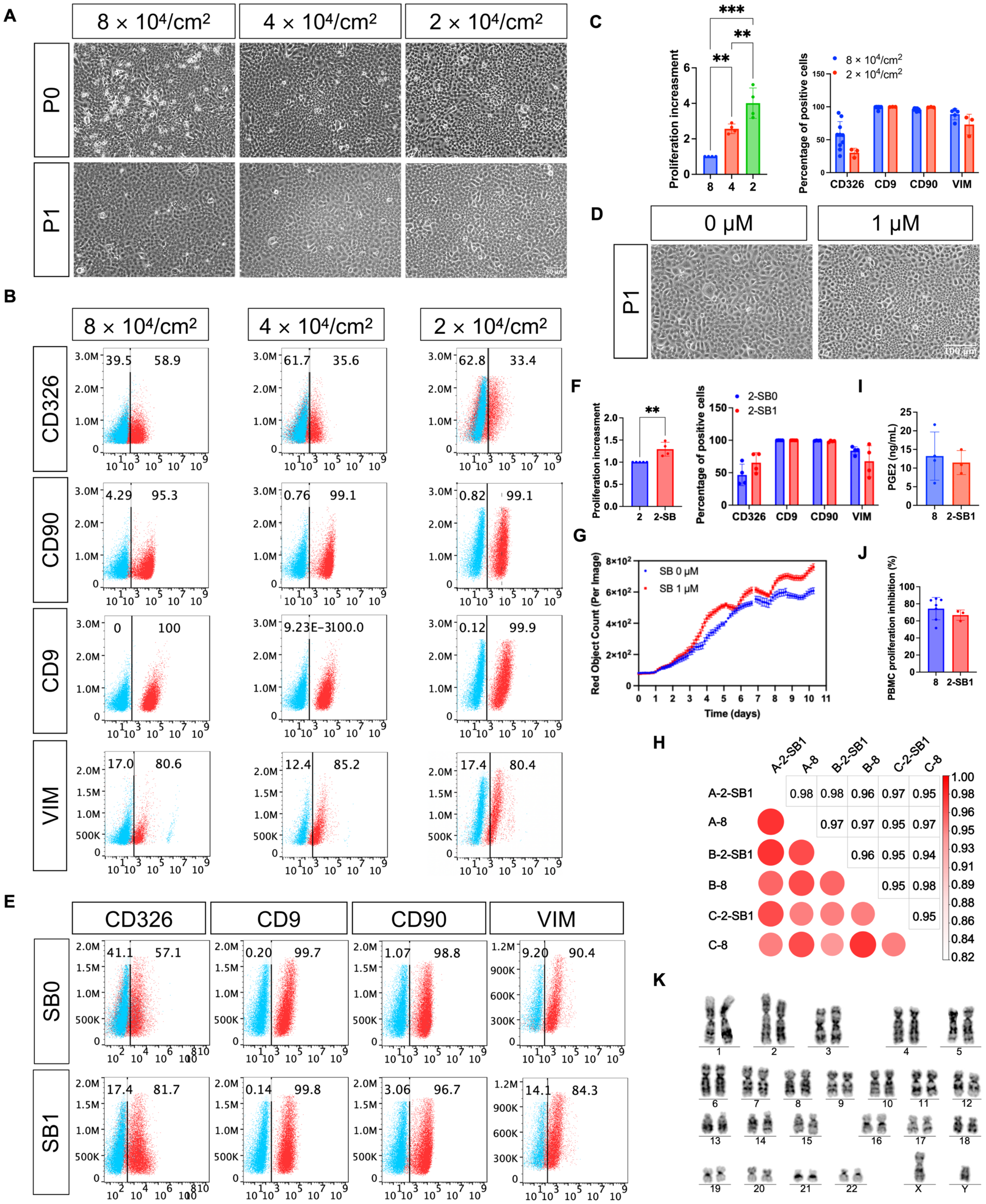

Figure S10. RNAseq analysis on hAECs from passages PreP0, P1-2-SB1 (hAECs of passage P1 cultured under low cell density (2 $\times 10^4$/cm^2) and condition-defined serum-free MQ3 medium), and P1-8 (hAECs of passage P1 cultured under high cell density (8 $\times 10^4$/cm^2) and MQ2 medium).

A. Expression heatmap of genes in the *cellular senescence* (GO) pathway in cultured cells (P1-2-SB1 and P1-8) and primary (PreP0) cells.

B. Expression heatmap of genes in the *epithelial cell proliferation* (GO) pathway in cultured cells (P1-2-SB1 and P1-8) and primary (PreP0) cells.

C. GSEA results of *response to stress* (GO) pathway between P1-2-SB1 and P1-8 hAECs.

D. KEGG enrichment analysis of differentially expressed genes between hAECs of P1-2-SB1 and P1-8.

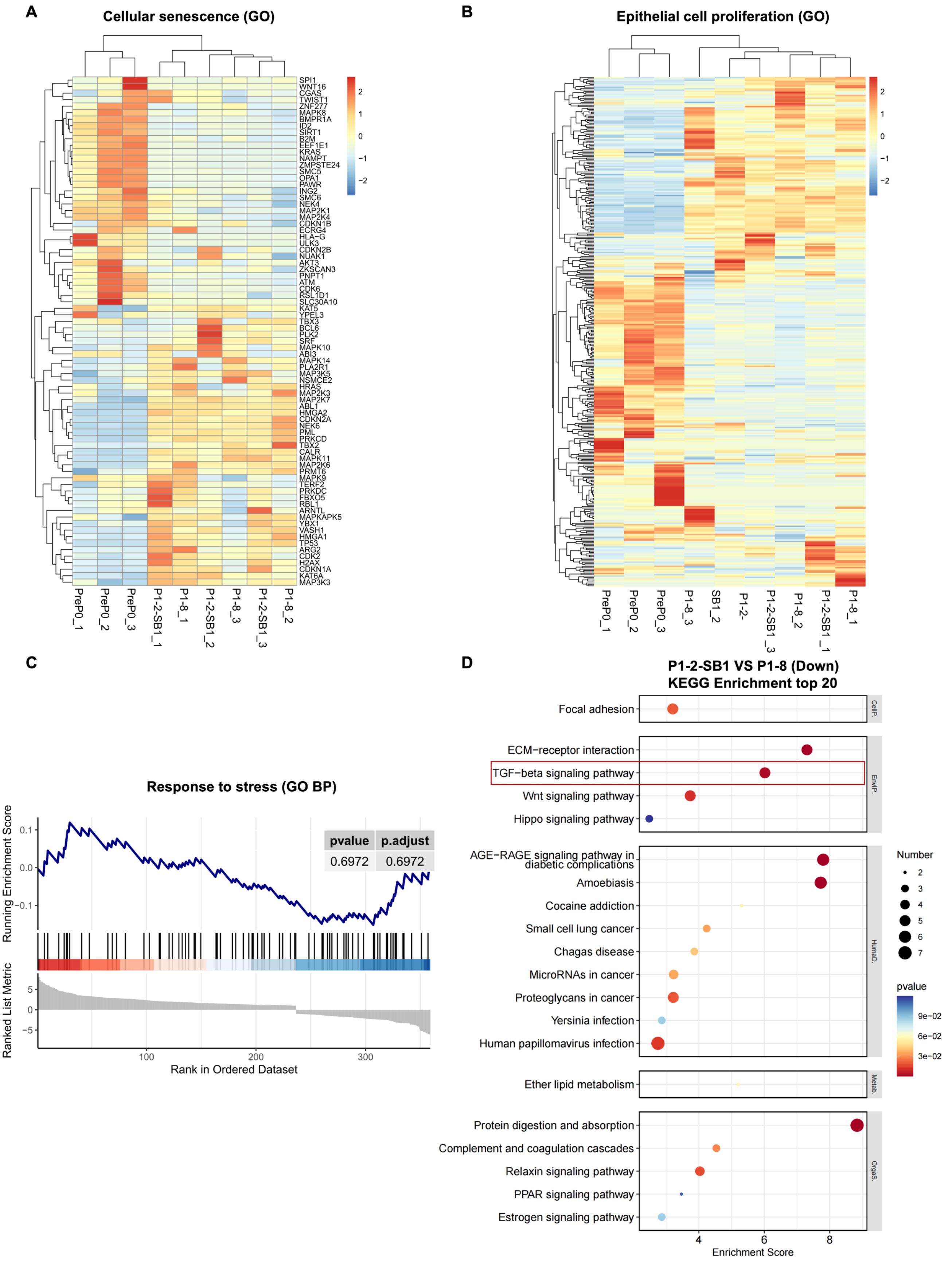
A
Cellular senescence (GO)
SPI1
WNT16
CGAS
TWIST1
ZNF277
MAPK8
BMPR1A
ID2
SIRT1
B2M
EEF1E1
KRAS
NAMPT
ZMPSTE24
SMC5
OPA1
PAWR
ING2
SMC6
NEK4
MAP2K1
MAP2K4
CDKN1B
ECRG4
HLA-G
ULK3
CDKN2B
NUAK1
AKT3
ZKSCAN3
PNPT1
ATM
CDK6
RSL1D1
SLC30A10
KAT5
YPEL3
TBX3
BCL6
PLK2
SRF
MAPK10
ABI3
MAPK14
PLA2R1
MAP3K5
NSMCE2
HRAS
MAP2K3
MAP2K7
ABL1
HMGA2
CDKN2A
NEK6
PML
PRKCD
TBX2
CALR
MAPK11
MAP2K6
PRMT6
MAPK9
TERF2
PRKDC
FBXO5
RBL1
ARNTL
MAPKAPK5
YBX1
VASH1
HMGA1
TP53
ARG2
CDK2
H2AX
CDKN1A
KAT6A
MAP3K3
PreP0_1
PreP0_2
PreP0_3
P1-2-SB1_1
P1-8_1
P1-2-SB1_2
P1-8_3
P1-2-SB1_3
P1-8_2
B
Epithelial cell proliferation (GO)
PreP0_1
PreP0_2
PreP0_3
P1-8_3
SB1_2
P1-2-
P1-2-SB1_3
P1-8_2
P1-2-SB1_1
P1-8_1
C
Response to stress (GO BP)
Running Enrichment Score
pvalue
p.adjust
0.6972
0.6972
Ranked List Metric
Rank in Ordered Dataset
D
P1-2-SB1 VS P1-8 (Down)
KEGG Enrichment top 20
Focal adhesion
ECM−receptor interaction
TGF−beta signaling pathway
Wnt signaling pathway
Hippo signaling pathway
AGE−RAGE signaling pathway in diabetic complications
Amoebiasis
Cocaine addiction
Small cell lung cancer
Chagas disease
MicroRNAs in cancer
Proteoglycans in cancer
Yersinia infection
Human papillomavirus infection
Ether lipid metabolism
Protein digestion and absorption
Complement and coagulation cascades
Relaxin signaling pathway
PPAR signaling pathway
Estrogen signaling pathway
CellP
EnvIP
HumaD
Metab
OrgaS
Number
pvalue
9e−02
6e−02
3e−02
Enrichment Score

Figure S11. Microcarrier screening.

A. The adhesion efficiency of hAECs on CT1 and CT3 microcarriers. Data were presented as mean ± SD, and unpaired two-tailed student's t-test was used for the comparison. n ≥3， $p < 0.05$ (*), $p < 0.01$ (**), and $p < 0.001$ (***).

B. The morphology of passages P1, P2 and P3 hAECs on CT1and CT3 microcarriers.

C. The growth curves of hAECs on CT1 and CT3 microcarriers. Data were presented as mean ± SD, and two-way ANOVA was used for the comparison. n = 3， $p < 0.05$ (*), $p < 0.01$ (**), and $p < 0.001$ (***).

D. The expression results of CD326 in hAECs cultured on CT1 and CT3 microcarriers.

E. The statistical results of CD326 in hAECs cultured on CT1 and CT3 microcarriers. Data were presented as mean ± SD, and unpaired two-tailed student's t-test was used for the comparison. n ≥3， $p < 0.05$ (*), $p < 0.01$ (**), and $p < 0.001$ (***).

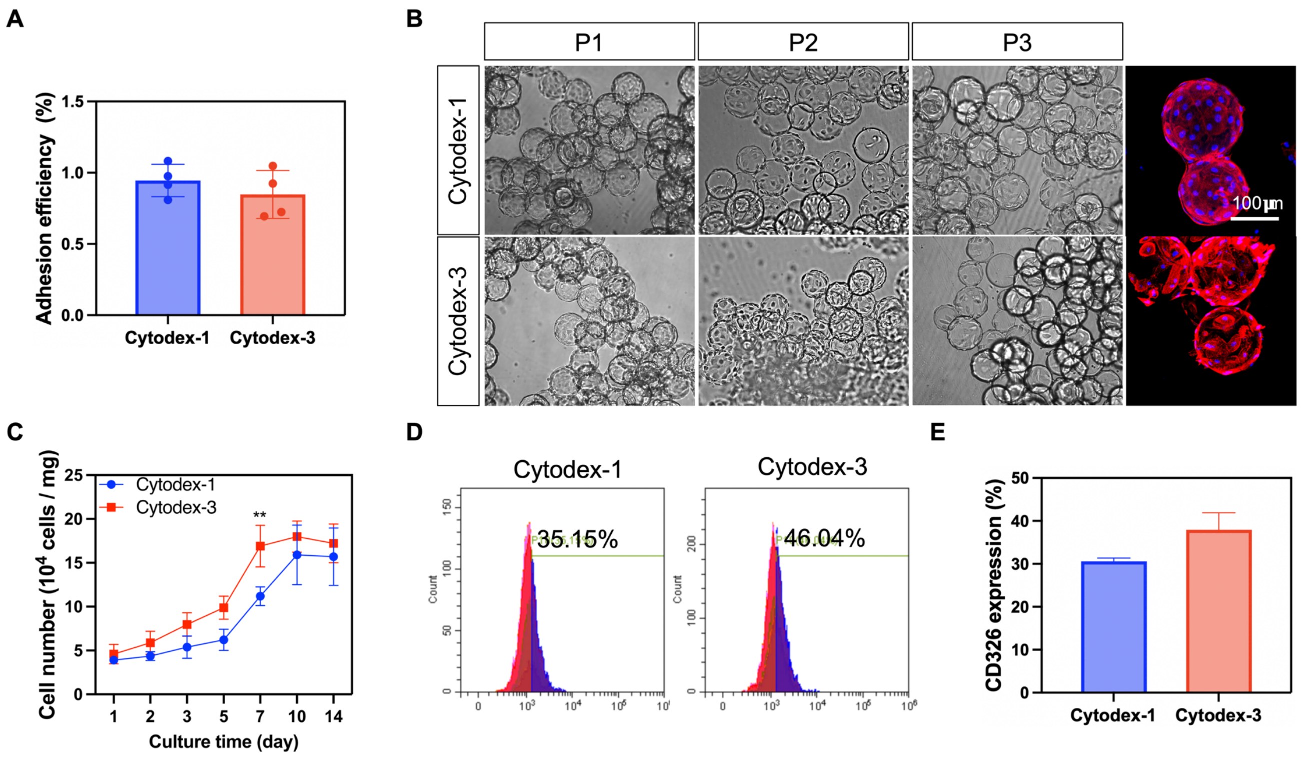

Figure S12. Specific validation of primers and antibodies.

A. Specific validation of primers used in qPCR. Data were normalized with the positive control group set as 1 and presented as mean ± SD, and unpaired two-tailed student's t-test was used for the comparison. n ≥3， $p < 0.05$ (*), $p < 0.01$ (**), and $p < 0.001$ (***).

B. Specific validation of antibodies used in flow cytometry.

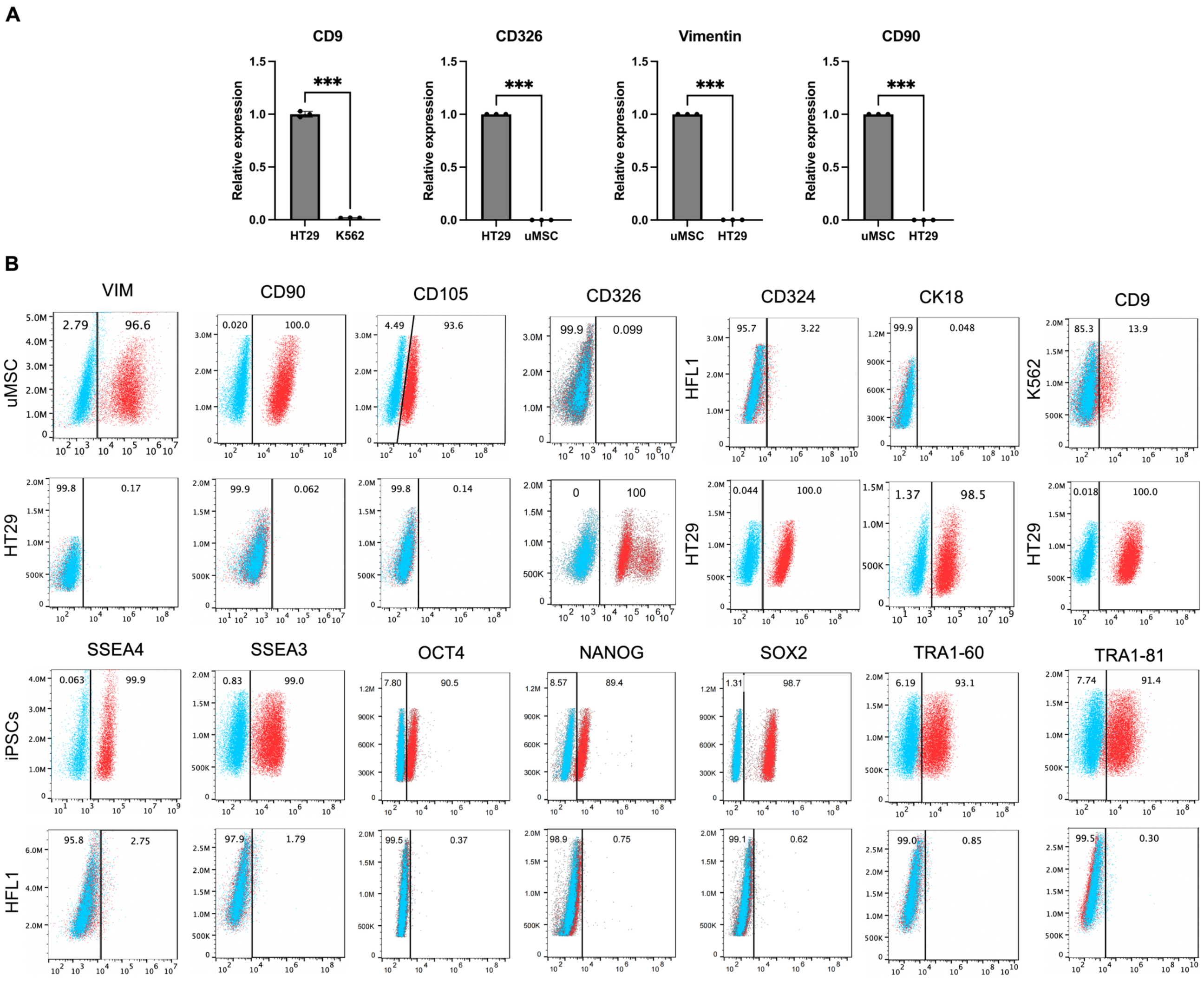

Table S1. The influence of SB431542, adding from passage P0, on the expression of epithelial and mesenchymal markers.

**CD326**	**P0**	**P1**	**P2**
**0 µM**	99.8	47	38.6
**0.5 µM**	99.9	68.3	57.8
**1 µM**	99.9	76.7	64.7
**2.5 µM**	99.9	87.5	77.3
**5 µM**	99.9	95.2	90.3
**CD9**	**P0**	**P1**	**P2**
**0 µM**	100	100	100
**0.5 µM**	99.9	100	100
**1 µM**	99.9	100	100
**2.5 µM**	99.9	100	100
**5 µM**	99.9	100	100
**CD90**	**P0**	**P1**	**P2**
**0 µM**	83.5	97.6	98.7
**0.5 µM**	63.5	89.6	94.4
**1 µM**	59.5	87	92.5
**2.5 µM**	53.6	85.4	92.6
**5 µM**	52	88	93.8
**VIM**	**P0**	**P1**	**P2**
**0 µM**	91.7	93.4	78.8
**0.5 µM**	87.9	83.5	57.4
**1 µM**	75.3	82.7	47
**2.5 µM**	58.1	54.9	40.3
**5 µM**	51.1	70.3	53.4

Table S2. Effect of SB431542, adding from passage P0, on the cumulative amplification fold of hAECs.

Cumulative amplification fold	P0	P1	P2
**0 µM**	3.381	8.132	12.492
**0.5 µM**	3.769	9.602	20.937
**1 µM**	4.089	10.983	24.804
**2.5 µM**	3.703	12.466	32.204
**5 µM**	4.458	12.533	27.329

Table S3. The influence of SB431542, adding from passage P1, on the expression of epithelial and mesenchymal markers.

**CD326**	**PreP0**	**P0**	**P1**	**P2**	**P3**	**P4**
**0 µM**	48.8	99.8	50.3	36.7	27.6	50.5
**1 µM**			83.3	64.3	51.2	23
**2.5 µM**			86.2	72.9	65.6	36.1
**CD9**	**PreP0**	**P0**	**P1**	**P2**	**P3**	**P4**
**0 µM**	99.4	100	100	99.9	99.9	99.3
**1 µM**			100	100	100	99.9
**2.5 µM**			100	99.9	100	100
**CD90**	**PreP0**	**P0**	**P1**	**P2**	**P3**	**P4**
**0 µM**	1.74	77.3	94.7	99	99.2	97.8
**1 µM**			88.4	96.5	96.4	90.7
**2.5 µM**			88.7	97.1	97	88.9
**Vimentin**	**PreP0**	**P0**	**P1**	**P2**	**P3**	**P4**
**0 µM**	29.7	59.8	56.4	60.6	77.2	91.7
**1 µM**			25.3	21	27.6	23.9
**2.5 µM**			23.3	23.2	22.7	13.6

Table S4. Effect of SB431542, adding from passage P1, on the cumulative amplification fold of hAECs.

**Cumulative amplification fold**	**P0**	**P1**	**P2**	**P3**	**P4**	**总和**
0 µM	2.421	2.093	1.654	1.000	1.000	8.377
1 µM		2.482	1.964	1.404	1.270	21.037
2.5 µM		2.075	2.200	1.410	1.233	19.202

Table S5. Primer sequences of qPCR.

	Forward primer (5'-3')	Reverse primer (5'-3')
**Vimentin**	GAGAACTTTGCCGTTGAAGC	GCTTCCTGTAGGTGGCAATC
**CD90**	CGCTCTCCTGCTAACAGTCTT	CAGGCTGAACTCGTACTGGA
**CD105**	CGCCAACCACAACATGCAG	GCTCCACGAAGGATGCCAC
**CD324**	TGAAGGTGACAGAGCCTCTGGAT	TGGGTGAATTCGGGCTTGTT
**CD326**	AATCGTCAATGCCAGTGTACTT	TCTCATCGCAGTCAGGATCATAA
**CK18**	GTTGACCGTGGAGGTAGATGC	GAGCCAGCTCGTCATATTGGG
**CD9**	CCTGCTGTTCGGATTTAACTTCA	TGGTCTGAGAGTCGAATCGGA
**PCNA**	GCGTGAACCTCACCAGTATGT	TCTTCGGCCCTTAGTGTAATGAT
**TOP2A**	ACCATTGCAGCCTGTAAATGA	GGGCGGAGCAAAATATGTTCC
**MKI67**	ACGCCTGGTTACTATCAAAAGG	CAGACCCATTTACTTGTGTTGGA
**OCT4**	CTGGGTTGATCCTCGGACCT	CCATCGGAGTTGCTCTCCA
**NANOG**	TTTGTGGGCCTGAAGAAAACT	AGGGCTGTCCTGAATAAGCAG
**SOX2**	TACAGCATGTCCTACTCGCAG	GAGGAAGAGGTAACCACAGGG
**ACTB**	GCACCACACCTTCTACAATGAG	ACAGCCTGGATGGCTACGT

Table S6. Antibodies

Antibodies	Supplier name	Lot number	Clone name	Catalogue number
**OCT4 (FC)**	Biolegend	B351973	3A2A20	653706
**NANOG**	Biolegend	B363380	16H3A48	B363380
**SOX2**	Biolegend	B371361	14A6A34	B371361
**SSEA4 (FC)**	BD	2005674	MC813-70	560128
**SSEA3 (FC)**	Biolegend	B366969	MC-631	330306
**TRA1-60 (FC)**	Biolegend	B323081	TRA-1-60-R	330610
**TRA1-81 (FC)**	Biolegend	B371474	TRA-1-81	330706
**Vimentin (FC)**	BD	1278253	RV202	562337
**CD90 (FC)**	Biolegend	B371535	5E10	328108
**CD105 (FC)**	Biolegend	B314810	43A3	323206
**CD324 (FC)**	Biolegend	B367158	67A4	324108
**CD326 (FC)**	Biolegend	B348989	9C4	324208
**CK18 (FC)**	Abcam	1051849-4	C-04	ab52459
**CD9 (FC)**	BD	2010170	M-L13	555372
**CK18 (ICC)**	Abcam	GR3205534-2	—	ab52948
**Vimentin (ICC)**	Abcam	GR3394053-8	RV202	ab8978
**OCT4 (ICC)**	Santa Cruz	J0622/221116	IgG2b κ	sc-5279
**NANOG (ICC)**	Abcam	GR3424098-10	—	ab21624
**SOX2 (ICC)**	R&D	KOY0622061	—	AF2018
