# Supplementary material for: Scale‐Up of Human Amniotic Epithelial Cells Through Regulation of Epithelial‐Mesenchymal Plasticity Under Defined Conditions
Source: Adv Sci (Weinh). 2025 Jan 13;12(11):2408581. doi: 10.1002/advs.202408581 (PMC11923953; doi:10.1002/advs.202408581)
Supplement: Supplementary file 2 — Supplemental Video 1 [file ADVS-12-2408581-s001.pptx]

## Slide 1
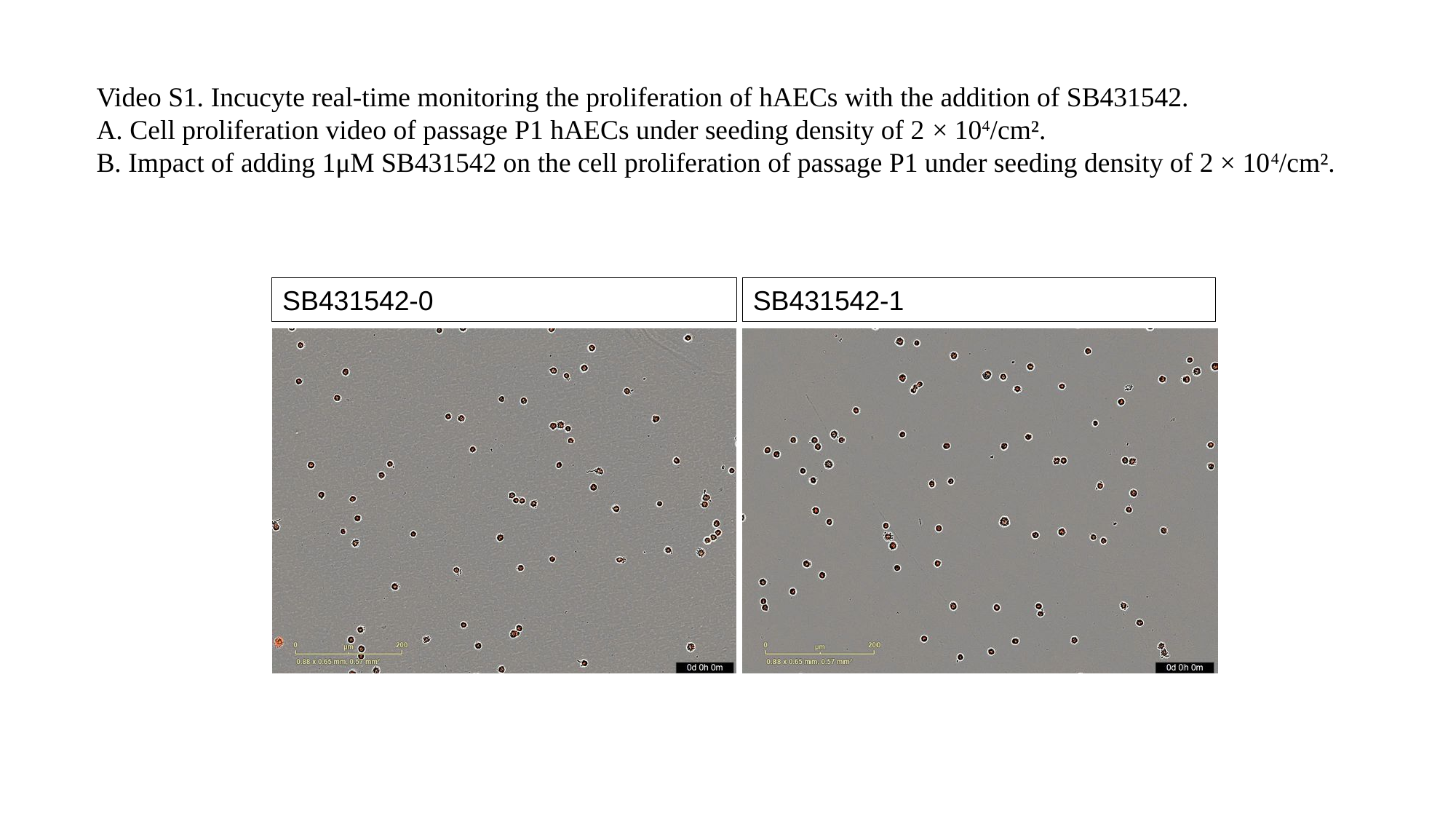

Video S1. Incucyte real-time monitoring the proliferation of hAECs with the addition of SB431542.
A. Cell proliferation video of passage P1 hAECs under seeding density of 2 × 10^4^/cm².
B. Impact of adding 1μM SB431542 on the cell proliferation of passage P1 under seeding density of 2 × 10^4^/cm².
SB431542-0
SB431542-1
